# Detection of invisible biological traces in relation to the physicochemical properties of substrates surfaces in forensic casework

**DOI:** 10.1038/s41598-024-63911-1

**Published:** 2024-06-10

**Authors:** Mathilde Recipon, Rémy Agniel, Philippe Kunemann, Arnaud Ponche, Franck Carreiras, Francis Hermitte, Johanne Leroy-Dudal, Sylvain Hubac, Olivier Gallet, Sabrina Kellouche

**Affiliations:** 1 Equipe de Recherche sur les Relations Matrice Extracellulaire‐Cellules, ERRMECe, (EA1391), Groupe Matrice Extracellulaire et Physiopathologie (MECuP), Institut des Matériaux, I-MAT (FD4122), CY Cergy Paris Université, Neuville Sur Oise, France; 2 Institut de Recherche Criminelle de La Gendarmerie Nationale, Cergy-Pontoise, France; 3 Institut de Science Des Matériaux de Mulhouse, Mulhouse, France

**Keywords:** Touch DNA, In vitro forensic models, Keratinocyte cells, Fingermarks, Substrate’ physicochemical characteristics, Adhesion/cytoskeleton proteins, Carbohydrate patterns, DNA, Skin models, Fluorescence imaging, Confocal microscopy, Scanning electron microscopy, Genotyping and haplotyping, PCR-based techniques, DNA probes, Fluorescent dyes, Fluorescent proteins, Cellular imaging, Carbohydrates, DNA, DNA sequencing, Characterization and analytical techniques, Techniques and instrumentation

## Abstract

Touch DNA, which can be found at crime scenes, consists of invisible biological traces deposited through a person’s skin’s contact with an object or another person. Many factors influence touch DNA transfer, including the “destination” substrate’s surface. The latter’s physicochemical characteristics (wettability, roughness, surface energy, etc.) will impact touch DNA deposition and persistence on a substrate. We selected a representative panel of substrates from objects found at crime scenes (glass, polystyrene, tiles, raw wood, etc.) to investigate the impact of these characteristics on touch DNA deposition and detection. These were shown to impact cell deposition, morphology, retention, and subsequent touch DNA genetic analysis. Interestingly, cell-derived fragments found within keratinocyte cells and fingermarks using in vitro touch DNA models could be successfully detected whichever the substrates’ physicochemistry by targeting cellular proteins and carbohydrates for two months, indoors and outdoors. However, swabbing and genetic analyses of such mock traces from different substrates produced informative profiles mainly for substrates with the highest surface free energy and therefore the most hydrophilic. The substrates’ intrinsic characteristics need to be considered to better understand both the transfer and persistence of biological traces, as well as their detection and collection, which require an appropriate methodology and sampling device to get informative genetic profiles.

## Introduction

At the scene of an offence, investigators are confronted with traces of different kinds on a wide range of substrates. Visible traces such as unwashed blood can be collected and analyzed, but what about invisible traces such as touch DNA?

Touch DNA, which refers to a loss of biological material transferred from a donor to an object or another person during physical contact^1–3^, is a real challenge for officers who have to collect it using a best assumption. These biological traces are invisible to the naked eye, cannot be detected using the usual tools (polychromatic lights like Polilight®, or chemiluminescence like Luminol/Bluestar®), and consequently may be poorly collected, even though they represent the majority of traces analyzed in forensic laboratories^4^. The composition of touch DNA is still not clearly defined in the literature. According to Burill et al.^2^ these traces may come from shed skin cells (corneocytes) and other biological material (body fluids, saliva, nasal secretions, sebum, epithelial cells, etc.), but van Oorschot revealed as early as 1997 that it is possible to obtain genetic profiles from manipulated objects^5^.

Several factors influence the deposition of touch DNA, such as the nature of the contact (for example, the amount of DNA deposited increases with increasing finger pressure)^6^, the donor (the amount of DNA deposited may vary from one donor to another)^7,8^, and the surface specificities of the substrate on which the trace is deposited. In fact, depending on the type of substrate, the latter can have an impact not only on the deposition of the trace, but also on its persistence, detection, and collection^9^. For example, Moret et al*.*^10^ reported that the patterns of a digital trace were different depending on the roughness of the substrates. Belhadjamor et al*.*^11^ also found that on non-porous surfaces sweat-rich residues formed droplets, while sebum-rich residues formed a continuous film. Hughes et al*.*^12^ have shown that on low-hydrophobicity substrates there are more aqueous fingerprints (sweat), while on highly hydrophobic substrates there are more oil- and grease-rich residues. Hughes et al*.*^13^ also found that the topography of the deposit surface changes with the surface roughness of the substrate, indicating that the deposit conforms to the morphology of the substrate. The substrate-dependent trace may also persist differently. Kaelser et al.^4^ showed that substrates (textured rubber, untreated mild-steel tubing, cotton fabric) stored in a standard laboratory environment allowed touch DNA to persist for at least 9 months. On the other hand, when surfaces are outdoors, the persistence of touch DNA varies^4^ according to the type of substrate.

A few studies have focused on the detection of touch DNA as a function of surface substrate type. Champion et al*.*^14^ attempted to assess the relevance of detection using Diamond™ Nucleic Acid Dye (Promega, USA) on a range of substrates in order to provide recommendations on the use of Diamond. However, despite the positive effects of Diamond™^15,16^, negative interaction states during direct PCR have been reported^17^. In another study^18^, our team developed a proof-of-concept based on a set of markers for the optimal detection of touch DNA combining cellular targets (DNA, keratin, laminin) and molecular targets such as carbohydrate patterns present in human cell glycoproteins (mannose, galactose, N-acetylglucosamin, and fucose) in order to improve their subsequent collection and success of genetic profiling. This innovative proof-of-concept made it possible to detect touch DNA, notably on a mobile phone, but it remained to be tested on several types of substrate.

Further research into the physicochemical composition of substrates and their impact on the deposition, persistence, detection, and collection of touch DNA remains essential for the forensic world’s ability to enhance the editing of robust genetic profiles, allowing for better decision-making during criminal investigations in keeping with the 2020–2025 European Union Security Strategy for fighting organized crime. In this way, this study has attempted to respond to this challenge by:

I. Developing in vitro touch DNA models on a panel of crime-scene substrates: reconstruction of touch DNA from characteristic cells (keratinocyte cells derived from a skin and fingermark touch DNA model) on different substrates that mimic those typically found in the field.
II. Analysis of physicochemical characteristics on substrate surfaces likely to modulate the deposition and persistence of touch DNA biological traces.
III. Detection of biological traces on several substrates, their persistence in time, and obtaining informative DNA profiles after their collection.

## Results

### Development of in vitro touch DNA models

**Figure 1:**
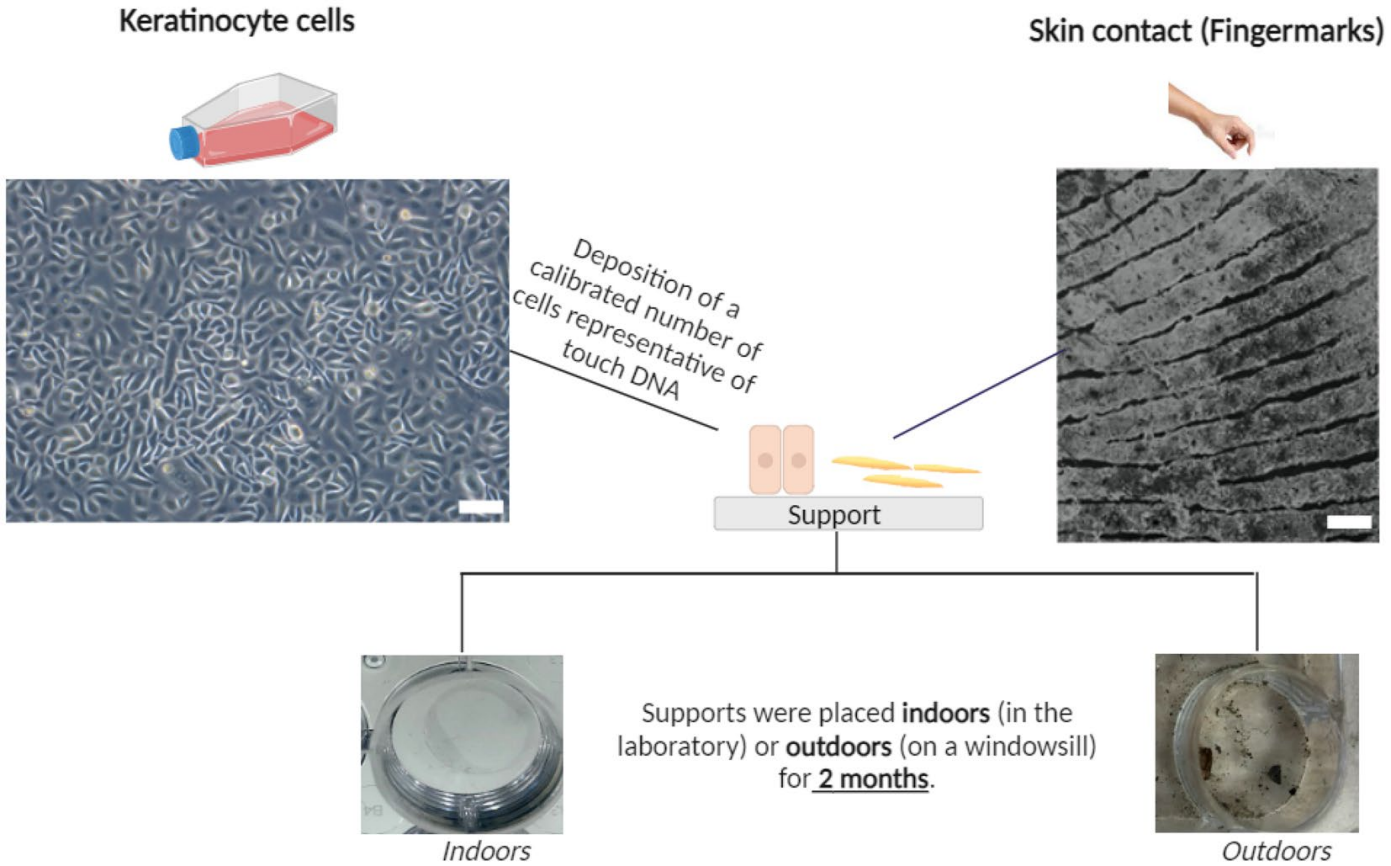
Representation of the in vitro models developed during this study. Keratinocyte cells or a fingermark were deposited on 10 substrates: glass, polystyrene, C1, C2, PVC floor covering, sticky adhesive tape, non-sticky adhesive tape, metal, varnished wood, and raw wood. In vitro models were then placed either indoors (in the laboratory) or outdoors (on a windowsill) for 2 months (Table 4). Scale bar for keratinocyte cells: 30 µm. Scale bar for fingermarks: 1 mm.

One of the aims of this study is to develop calibrated in vitro touch DNA models (i.e. mock samples) on different substrates. To this end, two types of in vitro models are proposed (Fig. 1):

1. As a proof-of-concept, we deposit a calibrated number of skin cells: keratinocyte cells. For those related to skin cell deposition, they are calibrated on the basis of a likely number of cells characteristic of those potentially found in touch DNA, seeded on substrates of defined typologies.
2. Throughout the study, we will compare this first in vitro model with the second one, which is a deposit of skin contact, namely fingermarks (superficial layers of the skin contain corneocytes^2,18,19^ which are anucleate (but with free DNA) keratinocyte cells in terminal differentiation), for 10 s on the substrates.

In the field, crime scenes are not necessarily found only indoors, they can also be outdoors. We therefore decided to take this parameter into account and expose the in vitro models indoors and outdoors for a certain period of time (2 months) (Table 4).

### Analysis of the global topography of the substrate surface

**Figure 2:**
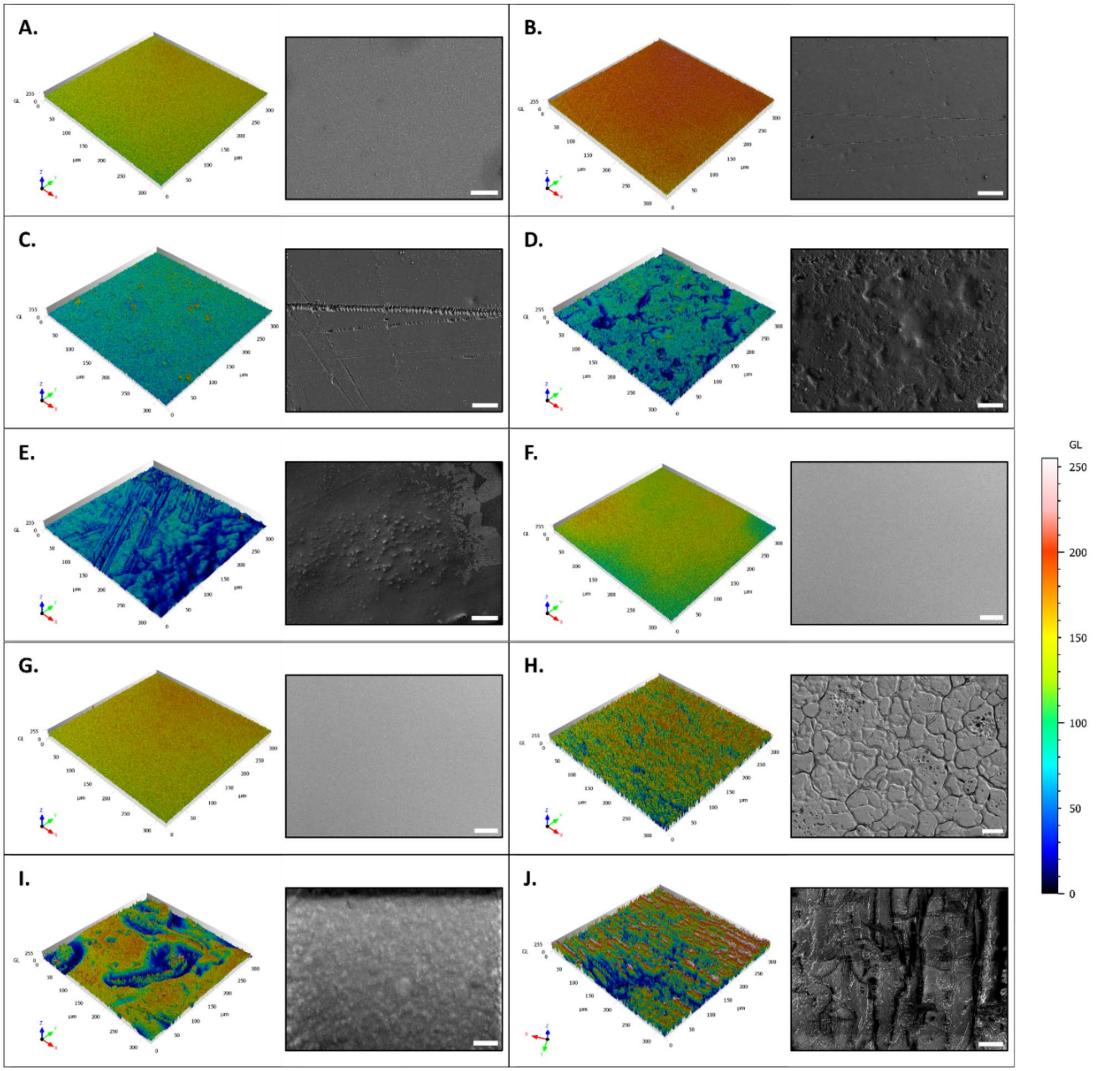
Analysis of substrate surface topography by scanning electron microscope and confocal microscopy. 10 substrates were analyzed by confocal microscopy imaging to obtain data on surface topographies: glass (**A**), polystyrene (**B**), C1 (**C**), C2 (**D**), PVC floor covering (**E**), sticky adhesive tape (**F**), non-sticky adhesive tape (**G**), metal (**H**), varnished wood (**I**), and raw wood (**J**). The surface of the substrates was first observed using SEM. For the adhesive tape, only one image was shown, as the images of the sticky and non-sticky adhesive tape are identical. Scale bar: 50 µm. The surface topography of the substrates was then analyzed by confocal microscopy. The scale [LUT rainbow: from valleys (blue) to peaks (red)] ranges from 0 to 250 µm.

In order to gain an initial insight into the surface topographies of the substrates, a scanning electron microscopy (SEM) approach was used for qualitative visual assessments. This was followed by a confocal microscopy analysis of the topography to illustrate the crepe pattern and variation of the various surfaces (Fig. 2).

On a microscopic scale (50 µm), image acquisitions by SEM showed that glass, polystyrene, and adhesive tape have flat and homogeneous surfaces. Whereas C1, C2, PVC floor covering, metal, and wood have flat but more heterogeneous surfaces. PVC floor covering and varnished wood, for example, had more granular surfaces, while C2 and metal had more or less deep valleys. On raw wood, on the other hand, the surface was a tangle of fibers.

The surface topography of the substrates analyzed by confocal microscopy showed that glass, polystyrene, C1, and adhesive tape (sticky/non-sticky) have a homogeneous crepe pattern with a relatively uniform structure. C2, PVC floor covering, metal, varnished wood, and raw wood had more heterogeneous surface topographies. C2 and PVC floor covering presented a topography with significant valleys but low peaks. For metal, raw wood, and varnished wood the topography showed the presence of numerous peaks and valleys. Raw wood had the highest peaks out of the whole panel.

There was therefore a good concordance, for each substrate, between SEM surface analysis and confocal microscopy topography.

### Analysis of substrate roughness, wettability, and surface energy

**Figure 3:**
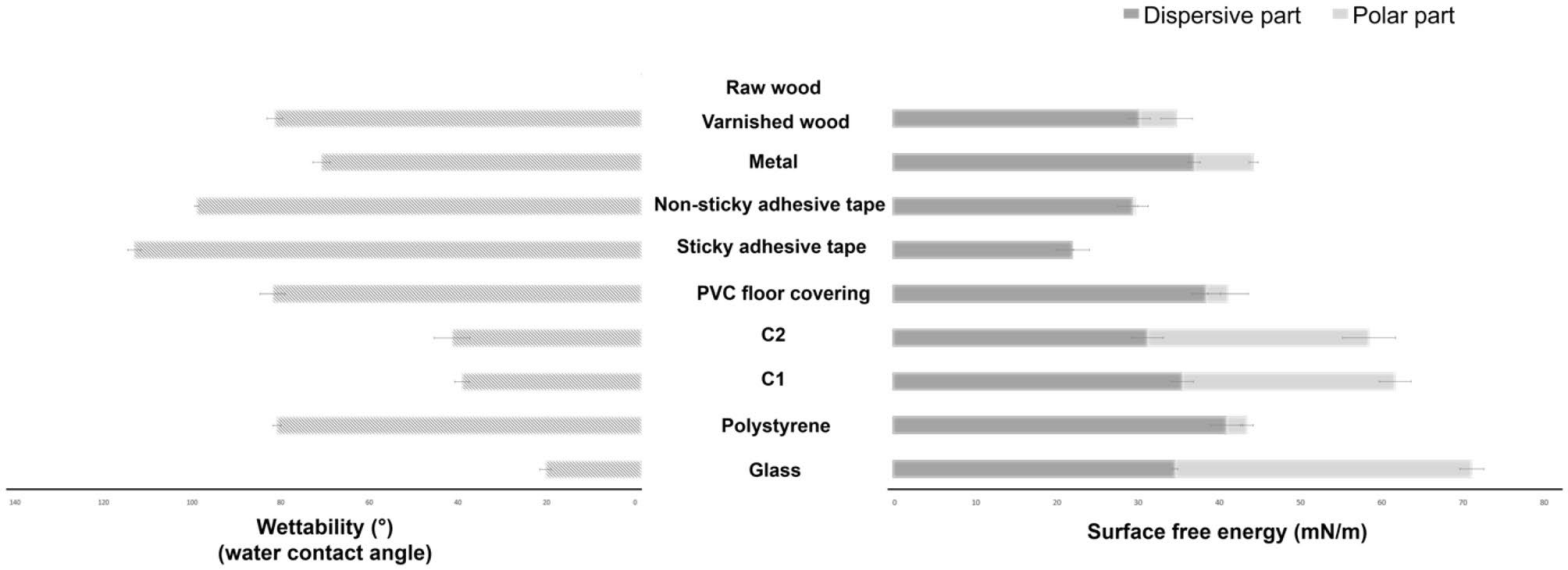
Wettability and surface energy measurements on substrates. Figure presented are the averages ± standard error (SE) of 5 wettability and surface free energy measurements made on the 10 substrates. This surface free energy is the sum of a dispersive part and a polar part. For raw wood samples with high porosity, wettability and surface free energy could not be measured due to the absorption of drops by the substrate.

**Figure 4:**
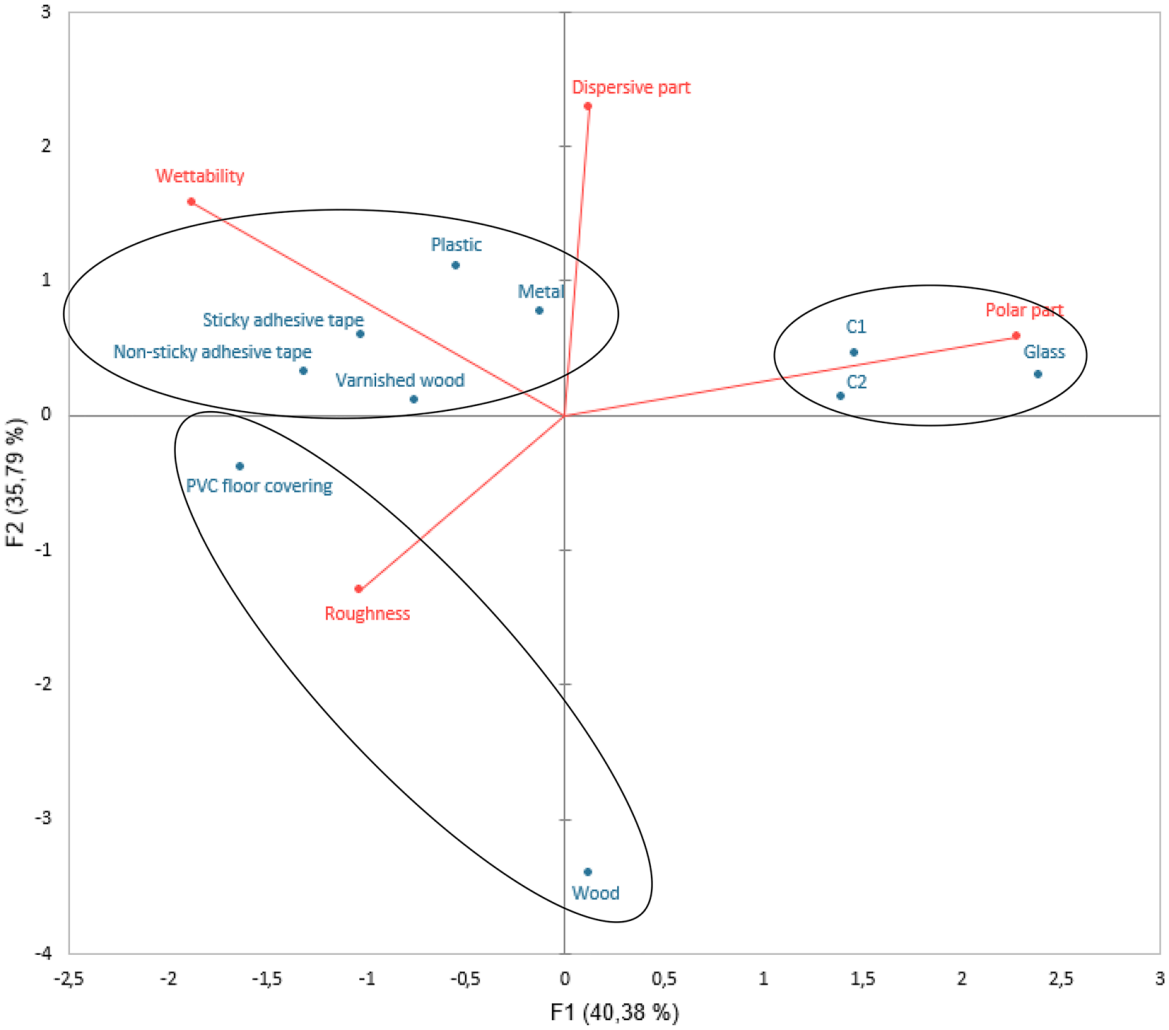
Principal component analysis of roughness, wettability, and surface free energy parameters. This graph represents the Principal Component Analysis (PCA) of roughness (Ra), wettability, and dispersive and polar parts. PCA is based on the specific variance of the three variables (roughness, wettability and surface free energy) and extracts a minimum number of factors (here two factors: F1 and F2) that explain the greatest possible proportion of the specific variance. The almost right angle formed by wettability and roughness indicates that these two variables are independent of each other.

A combination of physicochemical approaches has been used to characterize the substrate’s roughness, microstructure, and surface characteristics. First, roughness measurements were carried out to establish the different reliefs present on the surface of the substrates (Table 1, Fig. 4). Wettability measurements assessed the hydrophobic or hydrophilic dynamics of fluids as a function of substrate surface. Surface free energy measurements were carried out to determine the level of attractive forces within the substrate, which could enable interactions (hydrogen or Van der Waals) to take place between the substrate and the biological traces (Figs. 3, 4).

**Table 1:** Roughness measurements on substrates.

Substrates	R*a* (µm)	R*q* (µm)	R*t* (µm)
Glass	0.02 ± 0.01	0.02 ± 0.01	0.32 ± 0.03
Polystyrene	0.05 ± 0.01	0.06 ± 0.01	0.42 ± 0.02
C1	0.54 ± 0.10	0.64 ± 0.11	2.74 ± 0.32
C2	2.50 ± 0.13	3.17 ± 0.16	16.24 ± 0.89
PVC floor covering	31.67 ± 3.66	35.94 ± 3.30	122.79 ± 11.94
Adhesive tape (sticky)	–	–	–
Adhesive tape (non-sticky)	0.13 ± 0.02	0.16 ± 0.03	0.63 ± 0.11
Metal	0.11 ± 0.03	0.21 ± 0.08	2.33 ± 0.66
Varnished wood	3.79 ± 0.74	9.24 ± 4.68	25.84 ± 3.96
Raw wood	8.55 ± 1.27	15.73 ± 4.63	57.42 ± 6.82

Data presented are the averages ± standard error (SE) of 10 roughness measurements made on the 10 substrates. R*a* is calculated as the average roughness value of peaks and valleys. R*q* is calculated as the root-mean square deviation roughness of peaks and valleys. R*t* is calculated as the vertical difference between maximum peak height and valley depth. No roughness measurements could be made for sticky adhesive tape due to the sticky nature of the substrate, which could damage the device.

In terms of roughness R*a* and R*q* (Table 1), the smoothest element in the panel was glass as there was a significant difference between glass and polystyrene (P < 0.05). Metal and non-sticky adhesive tape had a similar roughness R*a* and R*q*, with no significant difference. It should be noted that these surfaces were smoother than C1, C2, and varnished wood (respectively ranked from smoothest to least smooth). For R*a*, significant differences were found between the three substrates (P < 0.05, p value C1–C2 < 0.0001, p value C2-varnished wood = 0.0337), while for R*q*, no significant differences were found between C2 and varnished wood. The roughest panel surfaces were PVC floor covering and raw wood. However, between these two substrates, a significant difference was noted (P < 0.05, p value < 0.0001), thus indicating that the roughest element in our panel was PVC floor covering.

As far as R*t* is concerned, a number of observations can be made. A significant difference (P < 0.05) was observed between all the substrates, indicating that each substrate presented different vertical values between peaks and valleys, except for non-sticky adhesive tape and polystyrene. These two substrates had significantly different roughnesses (R*a* and R*q*), but similar R*t* values. Thus, peak heights and valley depths were relatively similar for polystyrene and non-sticky adhesive tape. It’s also important to note that a significant difference was observed for R*t* (P < 0.05, p value = 0.0642) between metal and non-sticky adhesive tape. However, when compared with R*a* and R*q*, the roughnesses were similar and no significant differences were found. Thus, metal and non-sticky adhesive tape were similar in mean roughness, but metal had higher peaks and/or deeper valleys.

In terms of wettability (Fig. 3), the most hydrophilic substrate of the panel was glass followed by C1 and C2. No significant difference was observed between C1 and C2, indicating that these substrates have similar wettability. PVC floor covering and varnished wood were more hydrophobic, but there are no significant differences between them. Polystyrene and metal presented significantly different degrees of wettability (P < 0.05, p value = 0.0002), indicating that the metal was more hydrophobic than polystyrene. The most hydrophobic substrates in the panel were sticky adhesive tape and non-sticky adhesive tape. Nevertheless, it is important to note that a significant difference (P < 0.05, p value = 0.0088) was found between the two substrates. Thus, the most hydrophobic element in our panel was sticky adhesive tape.

With regard to surface free energy, each substrate was represented by its polar and dispersive components. Thus, glass is considered a polar surface due to water adsorption on silanol groups. For C1 and C2, despite a strong polar tendency, the dispersive component was retained for the surfaces of the substrates.

For polystyrene, PVC floor covering, sticky and non-sticky adhesive tape, metal, and varnished wood, the surfaces of the substrates were classified as predominantly dispersive (hydrophobic).

Principal component analysis (Fig. 4) enabled us to distinguish different groups based on physicochemical parameters such as roughness, wettability, and surface free energy:

- Group 1: The roughest substrates (PVC floor covering, raw wood). This group is more heterogeneous in terms of dispersion as wettability and polar and dispersive components could not be measured on raw wood due to porosity.
- Group 2: The most hydrophilic substrates, considered to be the most polar of the panel (glass, C1, C2).
- Group 3: Substrates with the highest hydrophobicity and dispersive surfaces (sticky adhesive tape, non-sticky adhesive tape, varnished wood, polystyrene, metal).

### Study of the persistence and detection of touch DNA on substrates

**Figure 5:**
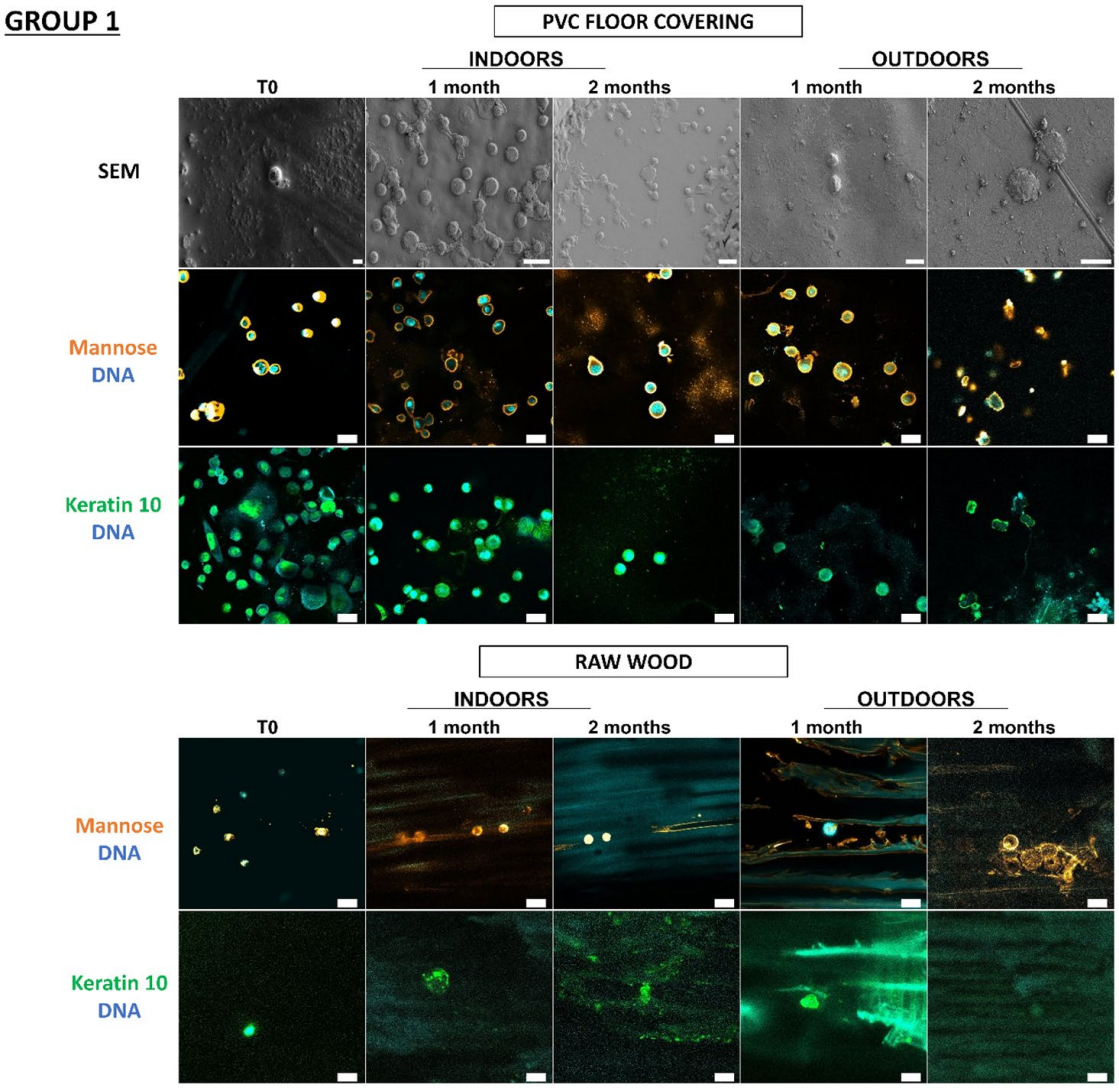

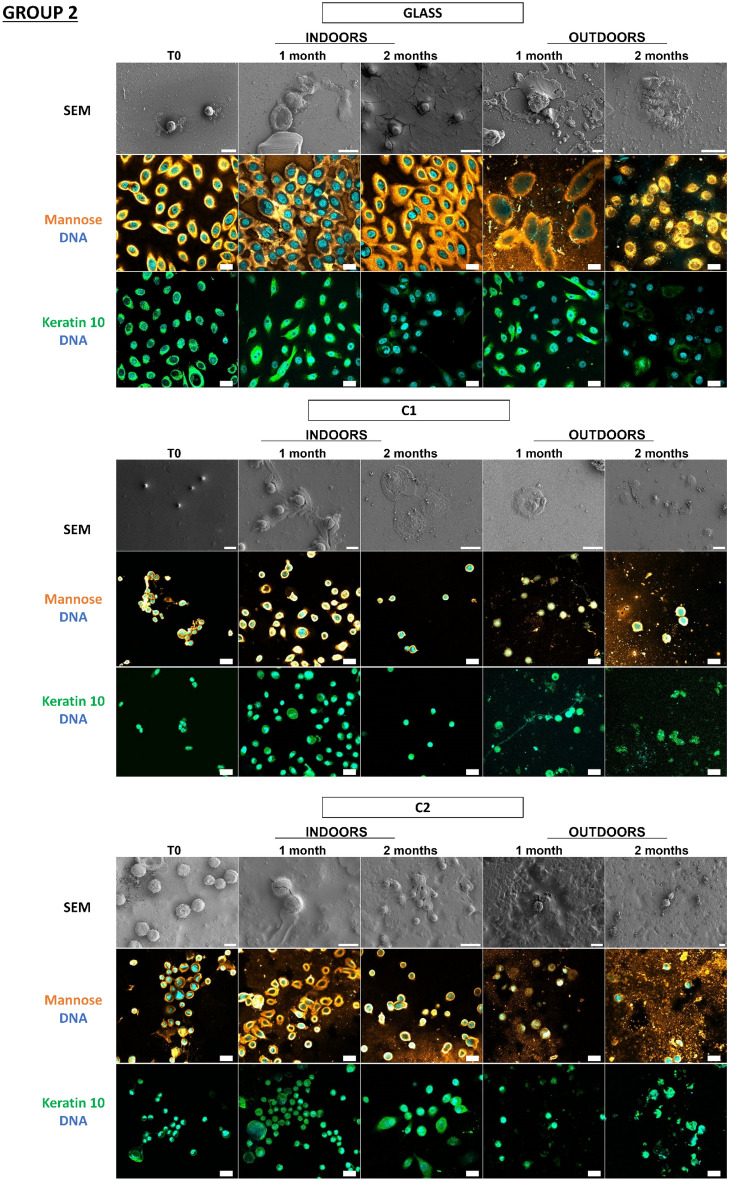

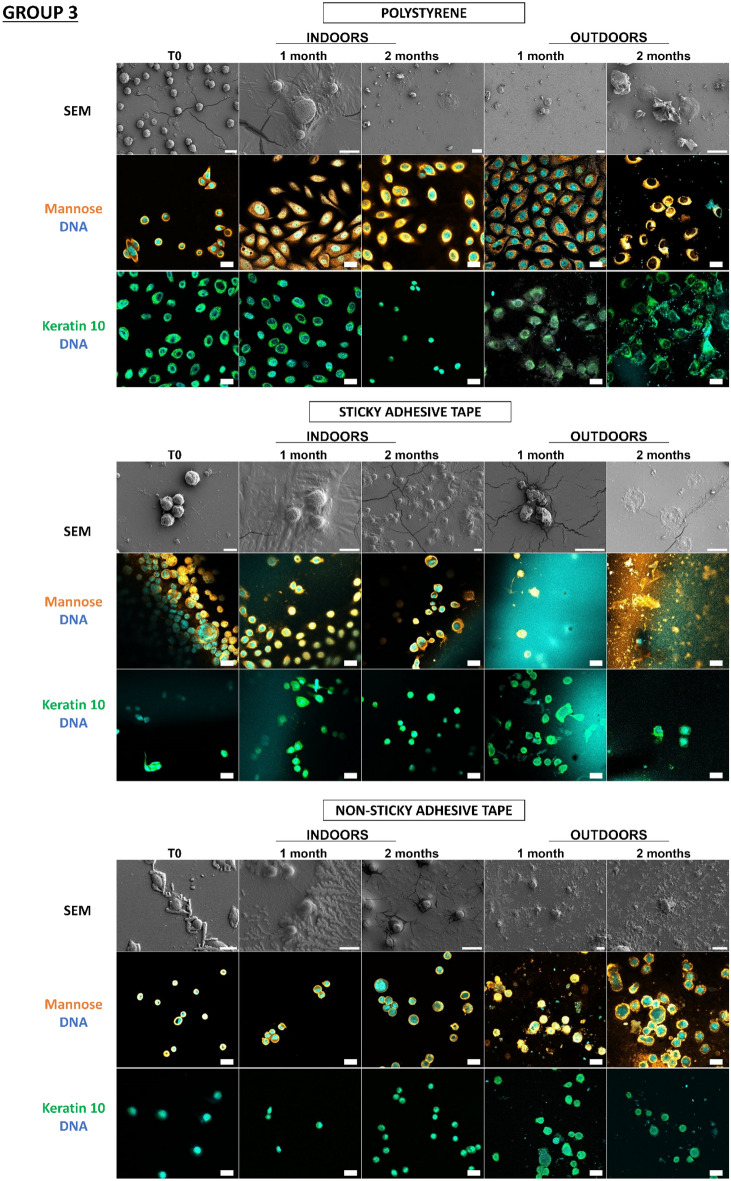

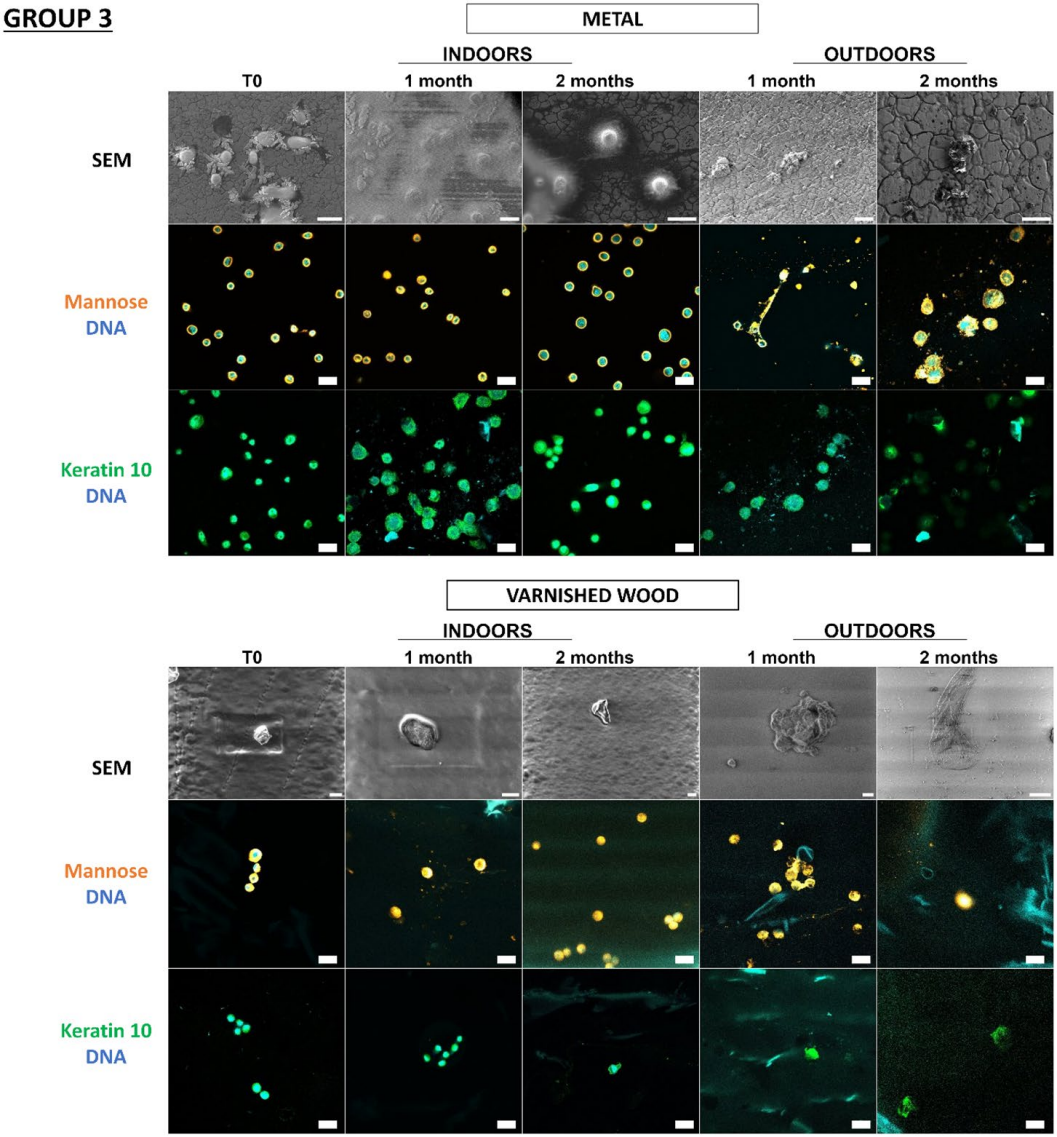
Detection and persistence of lectin, antibody, and DNA targets on keratinocyte cells on the panel of substrates. 5000 keratinocyte cells were seeded on 10 substrates. These 10 substrates were divided into 3 groups according to the principal component analysis of their physicochemical characteristics: group 1 (PVC flooring, raw wood), group 2 (glass, C1, C2), group 3 (polystyrene, sticky adhesive tape, non-sticky adhesive tape, metal, varnished wood). Substrates were placed indoors or outdoors for 2 months. The first line was the visualization of keratinocyte cells on substrates thanks to SEM. The second and third images showed keratinocyte cells visualized by confocal microscopy. For raw wood, no SEM images could be taken due to the fibrous nature of the substrate. Cells were incubated in the presence of Con A lectin (orange) that recognizes mannose carbohydrates, or labelled with antibody specifically directed against keratin 10 (Green). DNA from nuclei was stained with Hoechst 33342 (Blue). Images are representative data from three independent experiments. Scale bar: 10 µm.

**Figure 6:**
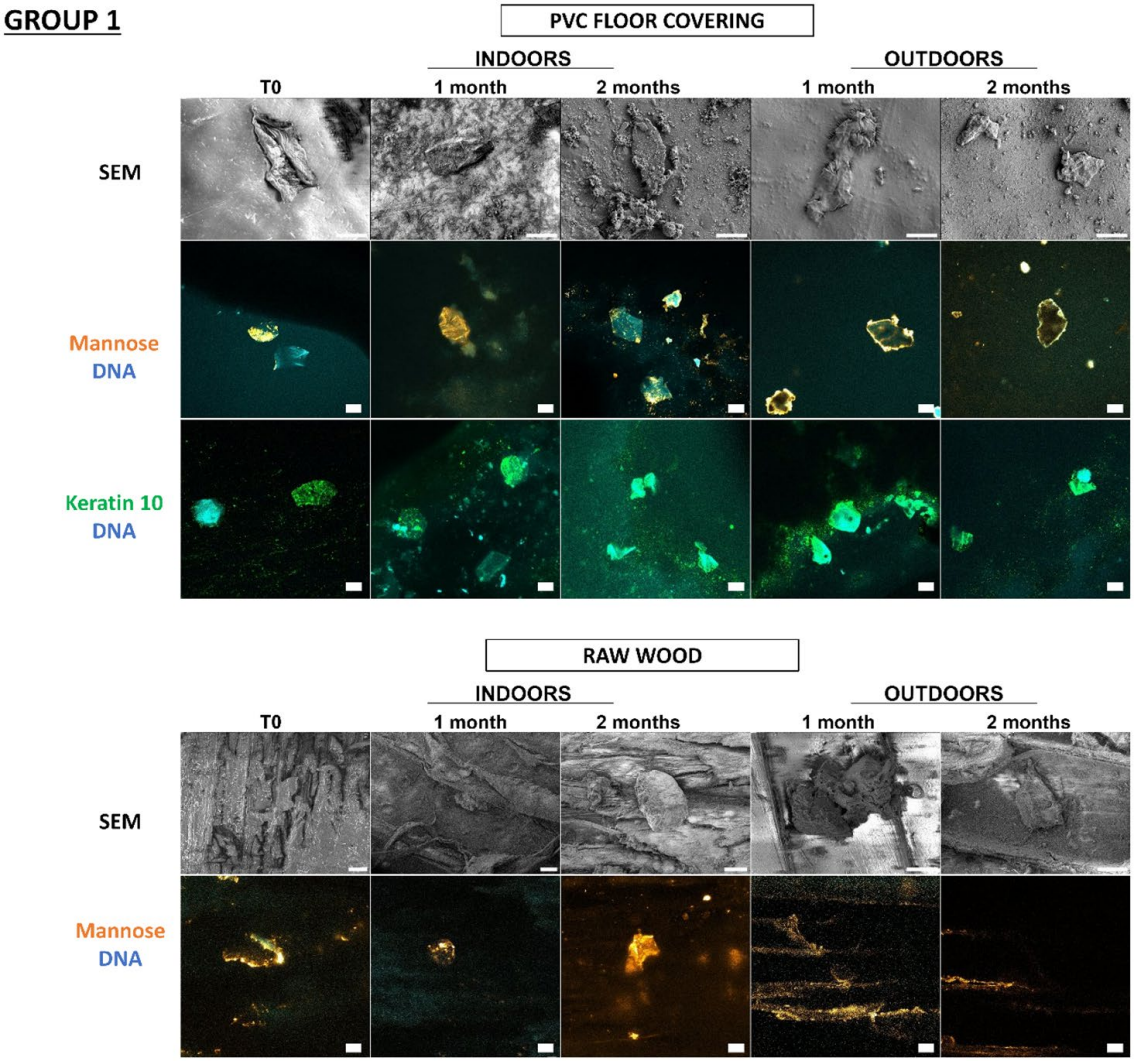

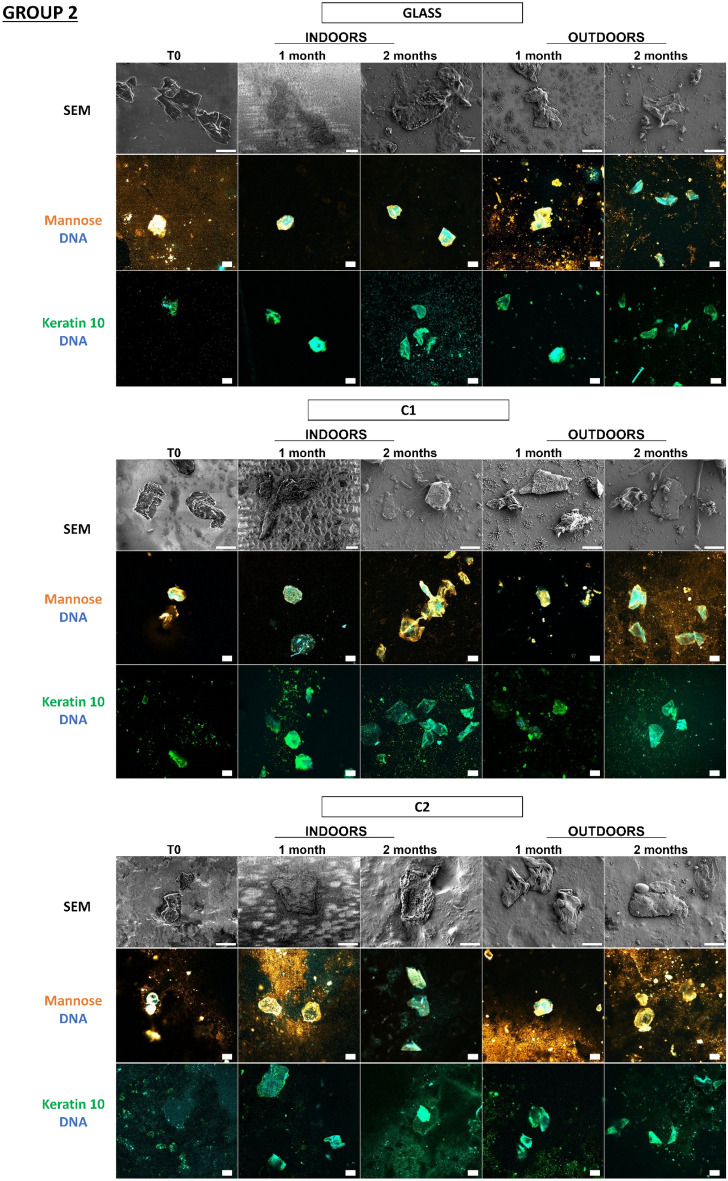

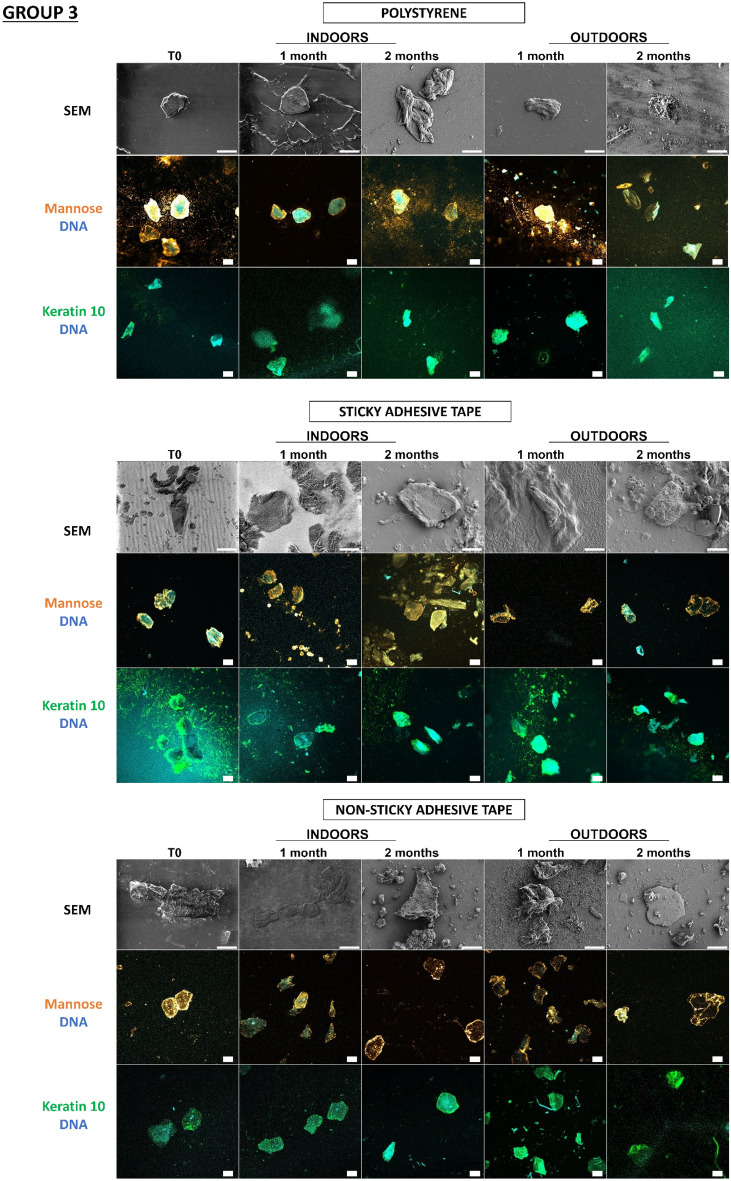

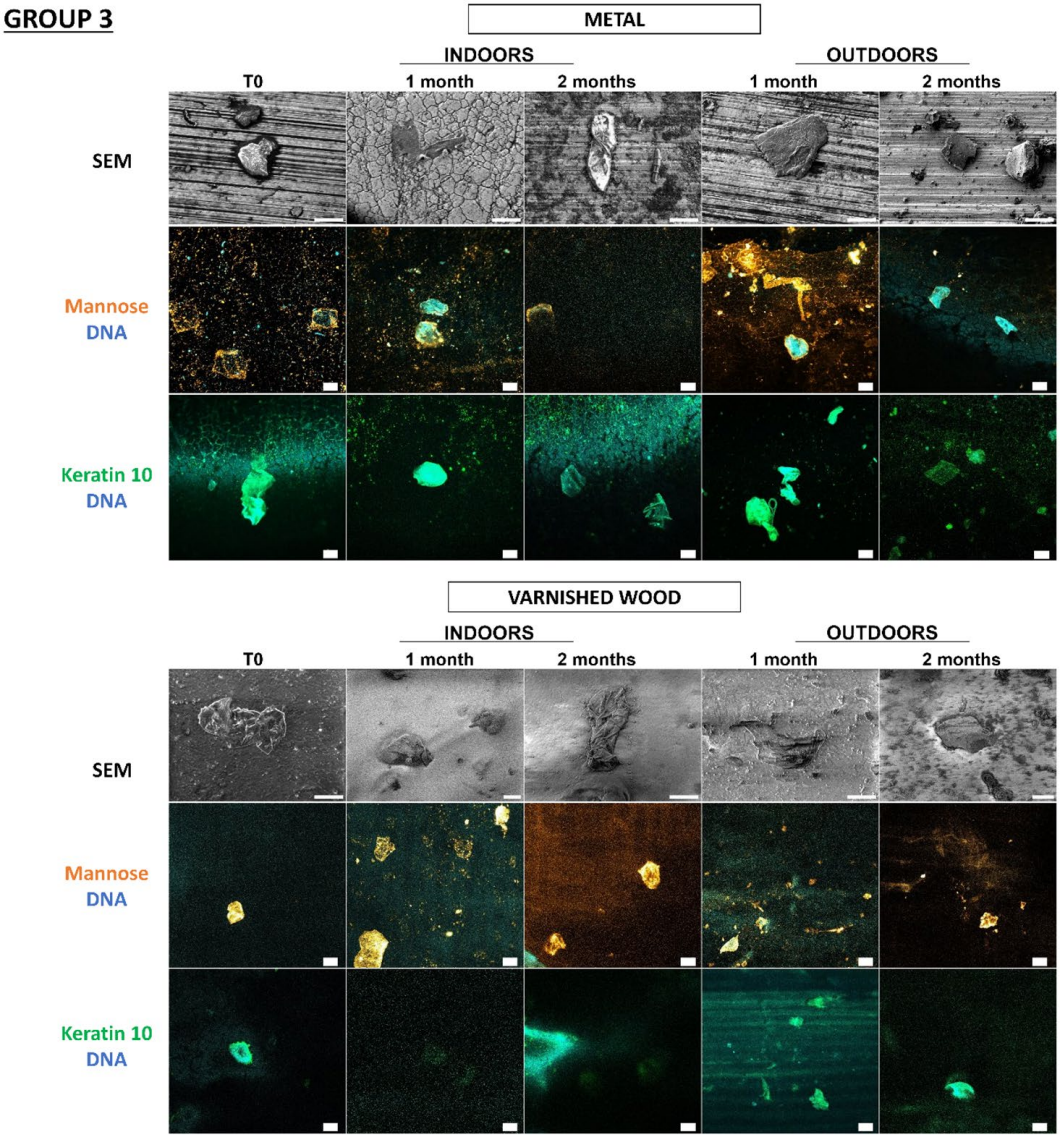
Detection and persistence of lectin, antibody, and DNA targets on fingermarks on the panel of substrates. Fingermarks were deposited on 10 substrates for 10 s. These 10 substrates were divided into 3 groups according to the principal component analysis of their physicochemical characteristics: group 1 (PVC flooring, raw wood), group 2 (glass, C1, C2), group 3 (polystyrene, sticky adhesive tape, non-sticky adhesive tape, metal, varnished wood). Substrates were placed indoors or outdoors for 2 months. The first line was the visualization of fingermarks on substrates thanks to SEM. The second and third images showed fingermarks visualized by confocal microscopy. Fingermarks were incubated in the presence of Con A lectin (orange) that recognizes mannose carbohydrates or labelled with antibody specifically directed against keratin 10 (K10, green). DNA was stained with Hoechst 33342 (blue). Images are representative data from three independent experiments. Scale bar: 10 µm.

In a previous study^18^, our team developed an original approach supported by proof-of-concept for detecting touch DNA on glass, targeting cell-derived fragments and, more specifically, proteins with adhesive structures (laminin, keratin 10) and carbohydrate moieties (mannose, galactose) in addition to DNA. Thanks to this approach, we wanted to test these various cellular and molecular targets for the detection of touch DNA on new types of substrates that are likely to be found on crime scenes. Indeed, many factors influence touch-DNA transfer, including the nature of the “destination” substrate surface. In the previous study, with the exception of glass, the other nine substrates were not tested for touch-DNA detection and not all substrates were characterized. Even though we focused on two targets presented here—mannose sequences (detected by Con A Lectin labelling) and keratin 10 (with antibodies directed against K10 labelling) (Figs. 5, 6)—all other targets (laminin, actin, etc.) were successful (Supplementary data, Figs. S1, S2). Thanks to this detection approach, we were able to investigate the biological traces’ persistence over time either through calibrated keratinocyte cell depositions or fingermarks.

In Fig. 5, thanks to scanning electron microscopy, we were able to see that the cells were present on all the substrates and could be visualized more than 2 months after deposition, both indoors and outdoors. Thus, at T0, keratinocyte cells showed classic morphological aspects that were more or less rounded depending on the type of substrate. For indoor conditions, for Group 1, for PVC floor covering, the cells remained round for 2 months. However, on raw wood, we were unable to find any cells due to the fibrous nature of the substrate. In Group 2, for glass and C1, the cells were round at T0, becoming more retracted and flattened over time, while for C2, the cells remained round over time. In Group 3, for adhesive tape, metal, and varnished wood, the cells remained round over time. Outdoors, whatever the group, the cells became increasingly flattened and thinned on the substrates, probably as a result of the unfavorable outdoor storage conditions.

We observe that for glass, polystyrene, C1, C2, PVC floor covering, sticky adhesive tape, non-sticky adhesive tape, and metal, it is possible to detect keratinocyte cells thanks to keratin 10 labelling, which was localized in the cytosol and near nuclei, and mannosyl residues, which are localized at the cell periphery. Both keratin 10 and mannose patterns persisted over time both indoors and outdoors. Surprisingly, DNA remains detectable after 2 months, even if the marking appears more diffuse. In the case of varnished wood and raw wood, the few cells could be identified, but identification was difficult due to the wood’s lignin auto-fluorescence.

So, despite the environmental conditions, the combinatorial-strategy detection deployed (the cellular and molecular targets) allowed the detection of keratinocyte cells on several possible substrates. We were able to observe that the morphological appearance of the cells changed over time depending on environment and substrate conditions.

In Fig. 6, we see that on all substrates the fingermarks that were still present could be detected thanks to the mannose carbohydrates, keratin, and DNA targets even after two months, indoors but also outdoors, irrespective of the group. The corneocytes present on the substrates had a flat morphology throughout the 2 months, indoors and outdoors, indicating an apparent conservation of the corneocytes over time. However, it should be noted that on raw wood corneocytes were difficult to localize due to the wood fibers giving a porous surface, so for keratin 10 labelling it was difficult to locate the corneocytes on several wood samples tested.

Despite unfavorable environmental conditions for its preservation, touch DNA can be detected specifically on most substrates, even over the long term, thanks to the cellular and molecular targets selected.

What is important to observe here is the complementarity of the labelling on the different substrates because a substrate would sometimes not allow detection while another would, especially in the case of raw wood. In conclusion, whatever the in vitro model (keratinocyte cells/fingermarks), the detection strategy is validated on a panel of substrates representative of objects found at a crime scene, and the substrates have no impact on the detection strategy.

### Genetic profile of collected touch DNA as a function of substrates

Two cell densities were deposited on each substrate. A low cell density (75 cells/cm^2^) was representative of DNA-poor traces^7,20^. A high cell density (250 cells/cm^2^)^21^ was representative of DNA standard traces. A comparison study was also made with a deposit of fingermarks on the substrates. The cells were then collected with a swab for genetic profile analysis.

**Figure 7:**
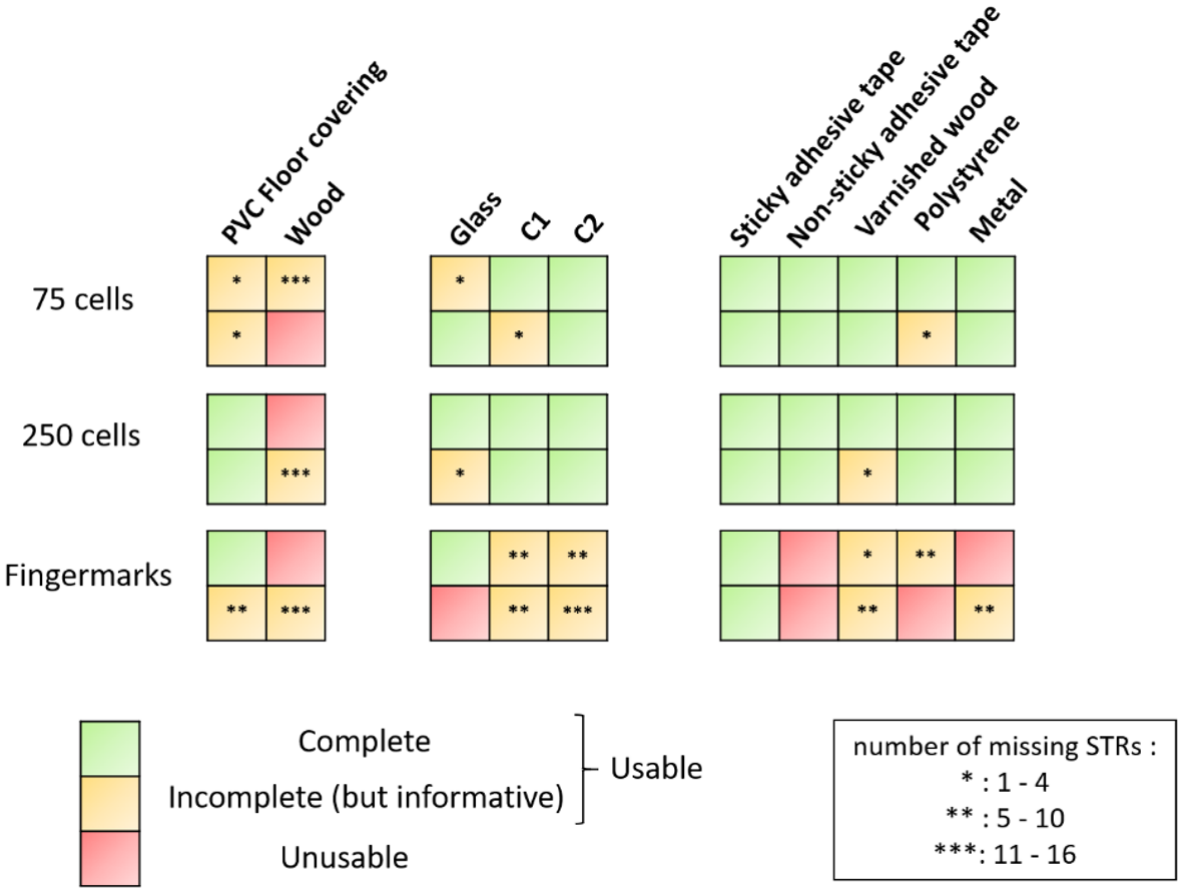
Genetic profiles according to substrates. Substrates were classified according to the 3 groups defined by the principal component analysis. In green, a profile is considered complete and usable. In yellow, a profile is considered usable but partial. And in red, the profile is considered unusable. A profile was usable if the number of complete Short Tandem Repeat (STR) markers was greater than 5, according to the requirements of the French database Fichier National Automatisé Empreintes Génétiques. A profile is defined as complete if all STR markers (22 STR) were amplified and present. Human gender determination was performed by analyzing the amelogenin gene which is common to both X and Y chromosomes. If the two quality sensors (Q and S) were visible on the electrophoregram, it meant that the PCR was successful and there was no inhibition. Results are representative of at least two independent experiments.

A certain variability of results was observed here (Fig. 7). All the genetic profiles (Supplementary data, Fig. S3) obtained expressed the internal Q/S controls, showing no PCR inhibition. For the same cell density, the responses of the substrates were heterogenous. But in general, taken together, the in vitro model with 75 cells provided the same response tendency as with fingermarks.

For glass and ceramics (C1, C2), the profiles were usable and partial, except for a glass duplicate which was unusable. Glass was the smoothest element, hydrophilic, and with the highest surface energy, whereas ceramic tiles 1 and 2 had a higher roughness.

We also note here that, for wood, which was one of the roughest and most hydrophilic substrates in the panel, the profiles were partial or unusable. This can be explained by the high porosity of raw wood, where biological material can infiltrate between the wood fibers and not be recovered during sampling. On the other hand, PVC floor covering, which was also one of the roughest elements but more hydrophilic than wood, showed usable profiles at all cell densities and for fingermarks. For sticky adhesive tape, used in the field to collect touch DNA, which was the most hydrophobic and had the least surface energy in the panel, all the profiles obtained were usable and complete (22 STR) for both cell densities and fingermarks. For varnished wood, polystyrene, and metal, the profiles obtained were more variable, particularly for the DNA-poorer fingermarks.

The impact of the physicochemical nature of the substrates should be considered as it can alter the accessibility of biological material and thus have an impact on genetic analysis.

## Discussion

The development of these in vitro touch-DNA models, using several on-field substrates, has enabled us to get insight into the impact of physicochemical characteristics on the editing of a genetic profile. These models have enabled to apply the proof-of-concept of touch-DNA detection using cellular-derived markers^18^ on a panel of substrates of different typologies.

Numerous forensic studies have traditionally used glass, polystyrene, or metal as standard substrates^22–24^. However, a few studies have been carried out on other types of substrate. These include Raymond et al.^22^, who observed buffy coat cells on window frames and pieces of leather; Cook et al*.*^23^, who studied blood, saliva, and sperm deposits on aluminum bars, iron, shoe soles, adhesive tape, and wood; and Tang et al.^24^, who analyzed buccal cells on a piece of steering wheel or a medicine box. Having taken into consideration the literature and actual crime scenes, we selected 10 types of substrate that are representative of materials found at a crime scene, namely: glass, polystyrene, two types of ceramic tiles C1 and C2, PVC floor covering, sticky adhesive tape, non-sticky adhesive tape, metal, varnished wood, and raw wood.

While the characterization of material surfaces is commonplace in the study of the cell colonization of biomaterials, it remains less explored in the forensic field, particularly in the study of cell–substrate interactions. The physicochemical characteristics and nature of a substrate influence cell deposition and behavior. It should be noted that, for the purposes of our study, the substrates were measured in their raw state in order to be as faithful as possible to crime scenes.

The physicochemical characterization enabled us to classify the substrates into three main groups:

- Group 1: Roughest substrates (PVC floor covering, raw wood).
- Group 2: Substrates with the highest surface energy and therefore the most hydrophilic (glass, C1, C2).
- Group 3: Substrates with the highest hydrophobicity and therefore the lowest surface energy (sticky adhesive tape, non-sticky adhesive tape, varnished wood, polystyrene, metal).

Once characterized, these substrates were tested by developing in vitro models of biological traces, which were similar to the reality in the field, by means of a calibrated deposition of cells characteristic of skin keratinocyte cells and fingermarks.

This choice of cell models was motivated by the fact that, as we saw in the introduction^19,25,26^, touch DNA derives notably from corneocytes desquamated from the surface of the skin. However, the latter are derived from keratinocytes that have undergone terminal differentiation, which leads to a flattened, anucleate cell morphology (with free DNA which could be used to edit a possible genetic profile), devoid of organelles and rich in keratin. It is for this reason that these cell models are best suited to our study. In the forensic literature, very few in vitro models have been developed using keratinocyte cells, except those in the study by Lee et al*.*^27^, which allowed them to study the persistence of DNA for up to 85 weeks on different substrates (drink containers, wristbands, paper, aluminum, glass, plastic). In vitro models are generally blood cell deposits^22^, mouth cells^24^, and sperm that are generally modelled. In our study, we were able to show that cells deposited on substrates of different typologies were present, could be detected using our detection strategy, and persisted over time for up to 2 months. Touch DNA detection was successful on most types of substrate. However, in the case of raw wood, the cells were difficult to detect as they may have penetrated into the fibers and no longer been on the surface. So, despite the heterogeneity of substrates, we have developed a universal detection strategy based on cellular-derived markers, where the type of substrate has no impact on this strategy. This detection strategy can be applied to a wide range of substrates, enabling touch DNA to be localized, better sampled, and optimized for genetic analysis.

In our previous study^18^, we showed that the detection strategy had no impact on the editing of a usable genetic profile. But here, depending on the number of cells deposited and the fingermarks on the different substrates, the genetic profiles obtained may not have been similar. In order to mimic traces of touch DNA “poor in biological material”, the density of keratinocyte cells had to be low on the substrates studied. Indeed, a trace is considered poor in DNA if it contains less than 500 pg (33 pg/microliter), or even very poor with less than 100 pg (6 pg/microliter)^1,20,28^. We chose 75 cells (corresponding to 450 pg DNA) because this corresponds to the low limit of the sensitivity threshold of the amplification kits for obtaining an exploitable and complete genetic profile. We were thus able to compare the genetic profiles of the keratinocyte cells with those of the fingermarks. However, the number of cells contained in fingermarks may be lower than our different cell densities. For example, according to Falkena et al.^29^, the number of cells in this study ranged from 0 to over 600 nucleated cells, with a median of 8.5. This study shows that cell numbers are relatively reliable, which could explain the quality of the different genetic profiles obtained.

In addition, physicochemical characteristics such as topography, roughness, wettability, and surface potentials are efficient key parameters for understanding the interaction with biological material and the quality of the genetic profile. For example, the porosity of materials can have a positive impact on adhesion and long-term cell retention. The wettability of substrates is also involved in cell behavior. Some authors^30^ have observed better cell adhesion and spreading on a hydrophobic substrate.

Our study demonstrates that both the rougher and more hydrophilic substrates tend to retain most of the deposited cells and therefore impact genetic analysis (except for PVC flooring, which is hydrophobic). On the contrary, both smooth and more hydrophobic substrates avoid more easily cell and biological material recovery. Presumably, on hydrophilic substrates, the number of cells transferred may be lower. Indeed, as stratum corneum cells are mixed with lipid cement (hydrophobic), the interaction may be higher on hydrophobic surfaces, so the number of corneocyte cells will be higher on hydrophobic than on hydrophilic surfaces.

Belhadjamor et al*.*^11^ have shown that, to maintain attractive forces between molecules and resist external forces, surface tension must be between 20 and 50 mM/m, i.e. a touch deposit will adhere to a substrate with a surface energy greater than 20 mM/m. In our panel, all substrates had a surface energy greater than 20 mM/m, which enabled the adhesion of touch DNA traces. However, Hughes et al. have established that one of the parameters influencing adhesion potential is surface energy. The lower the surface energy, the greater the substrate’s tendency to repel biological traces, and thus the lower the potential for deposit adhesion. This agrees with our study where we found that sticky adhesive tape had the lowest surface energy, but was the substrate for which all the genetic profiles were exploitable and complete. Cells are therefore less likely to adhere to low-energy surfaces, enabling better editing of a genetic profile.

Another parameter that could explain the heterogeneity of genetic profiles is collection. Indeed, collection depends on the type of substrate, the transfer of biological material onto the swab, and the person collecting. To optimize genetic analysis, we need to understand the link between physicochemical characteristics and the retention of biological material during collection. We were unable to investigate this aspect during this study, but work is currently underway in our laboratory to address this question (ANR 21-CE-0020, already funded by French (ANR) and Swiss (FNS) research agencies).

On the basis of our study, we can propose a universal detection strategy with a panel of markers that can be used on different types of substrate to generate usable genetic profiles. The study of the feasibility, stability, and durability of certain targets on different types of substrate is a new strategy in the forensic world, and opens news perspectives for the detection of touch DNA traces.

## Conclusion

This study has enabled us to develop in vitro touch DNA models, as close to reality as possible, in order to better understand the interactions between a substrate and a biological trace. We were thus able to show that it is important to consider physicochemical characteristics in order to understand the adhesion properties of the traces, and thus to collect them as effectively as possible in order to obtain a usable genetic profile. The physicochemical properties of the different substrates are essential not only to a better collection of traces, but also to explaining the qualitative and quantitative differences in the analysis of traces in justice.

Whatever the substrate, touch DNA could be located and detected thanks to a detection strategy based on cellular and molecular targets, without any impact from the nature of the substrate. This is a new strategy in the forensic world for the in vitro and in situ detection of touch DNA.

## Materials and methods

### Substrate selection

A representative panel of the different types of substrate likely to be found at a crime scene was selected by forensic experts (Table 2). The substrates had to correspond to the reality in the field, objects on which touch DNA could be deposited at a crime scene.

**Table 2:** Description of the general appearance of the various substrates used.

General appearance of substrates	Rigid	Flexible	Opaque	Translucent
Glass	✓	–	–	✓
Polystyrene (polystyrene)	✓	–	–	✓
Ceramic tile 1 (C1)	✓	–	✓	–
Ceramic tile 2 (C2)	✓	–	✓	–
PVC floor covering	✓	–	✓	–
Adhesive tape (sticky)	–	✓	–	✓
Adhesive tape (non-sticky)	–	✓	–	✓
316 L stainless steel (316L SS)	✓	–	✓	–
Varnished (polyurethane) wood	✓	–	✓	–
Raw wood	✓	–	✓	–

Ten substrates were chosen for this panel. For the tiles, two types were chosen: one visually smoother (C1) and the other rougher (C2). We chose not to work with fibrous materials (cotton fabrics, denim, etc.) as detection, collection approach, and genetic analysis are not the same as for substrate panels.

### Substrate pretreatment

The different substrates were immersed in a 70% ethanol solution, then rinsed with DNA-free water (grade 2) (Thermofisher, USA). The substrates were then placed in a petri dish and left to dry overnight before use. We chose this treatment for laboratory conditions in order to remove all particles from the surface of the substrates before starting our experiments. In the following experiments the substrates were left exposed to the open air and variations in climatic conditions, just like at a crime scene.

### Physicochemical characterization of substrate surfaces

#### Analysis of surface by scanning electron microscope

Substrates were metallized with 4 nm of platinum (ACE600, Leica), except for metal, before being imaged using a field emission gun scanning electron microscope (GerminiSEM 300, Zeiss, Germany) with an acceleration voltage of 2 keV under high or low vacuum. Secondary electrons were collected. Scan speed and line averaging were adjusted during observation. The data obtained were processed using Image J v2.1.0 software (National Institutes of Health, USA).

#### Analysis of topography by confocal laser scanning microscope

The topography of the substrates was analyzed by confocal microscope (LSM710, Zeiss, Germany) using a × 20 Plan-apochromat objective (NA: 0.8). A 640 nm laser was used for reflection with a z-depth of 100 µm. The images obtained were processed with Image J software version 2.1.0 (National Institutes of Health, USA) and ConfoMap® ST version 9 software (a derivative of MountainsMap® software, Digital Surf, France).

#### Profilometry

The profilometry of the surfaces studied was characterized using a Dektak 150 contact profilometer (Veeco, USA) (measurements carried out at the Laboratoire de Physicochimie des Polymères et des Interfaces, CY Cergy Paris Université). For each sample, an average surface profile was produced by measuring at ten different points along a 10 mm length with a stylus of 12.5 μm radius of curvature, applying a force of 3 mg to the stylus at 33 μm/s scan speed, and the various roughness parameters were calculated. Profiles were analyzed using the R*a*, R*q*, and R*t* parameters. Thus, R*a* corresponds to the difference between this deviation value and the central baseline. R*q* is calculated as the root-mean square deviation roughness of peaks and valleys. R*t* is calculated as the vertical difference between maximum peak height and valley depth.

#### Wettability and surface free energy

Wettability and surface free energy were measured by the Institut de Sciences des Matériaux de Mulhouse (France). These measurements were made using an optical tensiometer: Drop Shape Analysis System DSA 10 mk2 (KRUSS). A 2 µl drop of deionized water or diidomethane was placed on the sample surface and contact angles θ were measured. Measurements were made on both sides of the drop and were averaged. Only the angle of contact with water will be shown, because at a crime scene, investigators use water to moisten a swab before taking a sample from a substrate. Surface free energy was calculated using the Owens–Wendt–Rabel–Kaelble method (Table 3). The contribution of the probe liquids used was:

**Table 3:** Contribution of the probes used to calculate the surface free energy.

Substance	Polar (mN/m)	Dispersive (mN/m)
Water	51	21.8
Diiodomethane	0	50.8

For each sample, five measurements were taken at different points on the surface and the result presented was the average of the five measurements.

### Development of in vitro touch DNA models

#### Keratinocyte cells

A keratinocyte cell line (CCD 1106 KERTr CRL-2309TM) was isolated from human skin. This cell line was purchased from ATCC® (USA) with a certificate of conformity.

Cells were cultured in Keratinocyte-SFM medium with l-glutamine, supplemented with 2.5 ml bovine pituitary extract BPE (Thermofisher, USA) and 35 ng/ml recombinant human EGF (VWR, USA). Cells were grown in a humidified atmosphere at 37 °C and 5% CO_2_. To develop in vitro models, cells were seeded at various densities (75, 250, and 5000/cm^2^) on the substrates. Then, after 4 h incubation, the cell medium was removed and each substrate was left to dry for up to 2 months.

#### Skin contact samples: fingermarks

On each substrate, three different volunteers applied a fingermark for 10 s using moderate pressure^31^ on different days. Samples were left to dry for up to 2 months. All samples were obtained with the informed consent of the volunteers and the approval of the ethics committee of the Pôle Judiciaire de la Gendarmerie Nationale (PJGN), in accordance with the Helsinki Accords (1975) and the French National Charter of Research Integrity.

### Environmental conditions for in vitro models

To accurately replicate real crime scenes, all in vitro models (keratinocyte cells and fingermarks) were placed indoors in the laboratory and outdoors on a windowsill for 2 months (Table 4).

**Table 4:** Environmental conditions tested.

Samples	Indoors/outdoors	Temperature (average for 2 months)	Humidity (average for 2 months)
Keratinocyte cells	Indoors	19.0 ± 0.5 °C	50 ± 2%
	Outdoors	24.5 ± 3.3 °C	53 ± 5%
Fingermarks	Indoors	19.0 ± 0.5 °C	50 ± 3%
	Outdoors	24.5 ± 3.3 °C	53 ± 5%

### SEM analysis of in vitro models

All in vitro models were observed, at each time point (T0, 1, and 2 months), using a field emission gun scanning electron microscope (GerminiSEM 300, Zeiss, Germany). The same settings were used as for SEM surface analysis of the substrates.

### Proteins, carbohydrate patterns, and DNA fluorescence staining

For all in vitro models, an area of 1 cm^2^ was calibrated for depositing the biological material. At each time point (T0, 1, and 2 months), non-specific sites of keratinocyte cells and fingermarks were blocked 30 min with PBS-BSA 0.5% at room temperature and incubated for 2 h with primary antibody Keratin 10 (SAB4501656, (Sigma-Aldrich, USA), 1:50 in PBS-BSA 0.5%), or incubated for 20 min in a humidity chamber using a biotinylated lectin solution Con A (B-1005 (Vector laboratories, USA), at final concentration 10 µg/ml). After washes with PBS, substrates were incubated with fluorescent antibodies: Alexa Fluor 488 conjugated anti-rabbit antibody (A11008, Life Technologies, USA) during 1 h, or were incubated with Alexa Fluor 555 Streptavidin (S21381, Thermofisher, USA), diluted 1:600, for 20 min. Then, samples were stained 30 min with Hoechst 33342 (H1399, Thermofisher, USA) diluted 1:3000. Substrates were mounted with Prolong mounting medium (P36930, Invitrogen, USA). Controls without primary antibodies were negative.

### Microscopy analysis

Fluorescent labelling was observed with a confocal microscope (LSM710, Zeiss, Germany) equipped with a × 40 Plan-apochromatic oil immersion objective (NA: 1.2). The pinhole aperture was set at one Airy Unit (AU). Acquisitions were performed under the same conditions. The excitation wavelength for Hoechst 33342 was set at 405 nm and collected between 421 and 517 nm. The excitation wavelength for Alexa Fluor 488 was set at 488 nm and collected between 494 and 552 nm. The excitation wavelength for Alexa Fluor 555 Streptavidin was set at 561 nm and collected between 565 and 680 nm. The different analyses were conducted using Zen 2010 BSP1 software (Zeiss, Germany). Image acquisitions were processed with Image J software version 2.1.0 (National Institutes of Health, USA).

### DNA profiling

#### Collection from cell and fingermark samples

After deposition, keratinocyte cells or fingermarks were collected from the in vitro touch DNA models using a specific 1 mm^2^ collecting swab device routinely used by French Gendarmerie (microFLOQ® Direct swabs, Copan, Italy). Before collection, each swab was moistened with 1 µl of deionized water (grade 2). Then swabs were left to dry for 15 min at room temperature before the direct DNA amplification step. These swab devices contain all the chemicals required for cell lysis for further direct PCR amplification.

#### DNA amplification

The head of microFLOQ® was broken directly into PCR strip tubes. In each tube, 15 µl of molecular grade water and 10 µl of PCR mix were added. DNA amplification was done with Investigator 24plex QS (382415, Qiagen, USA). Investigator 24plex kits were used for multiplex amplification of CODIS (Combined DNA Index System) core loci, ESS (European Standard Set) markers, as well as SE33, DYS391, and Amelogenin markers. In addition, each kit contained internal PCR controls (quality sensors Q/S) for determining PCR efficiency. DNA amplification was performed with an Applied Biosystems™ Veriti™ Thermal Cycler (Applied Biosystems™ 4375305 from Thermofisher, USA) for 28 cycles according to the manufacturer’s protocol.

#### Genotyping

Using an ABI 3500xL genetic analyzer (Thermofisher), samples were genotyped. At the end of electrophoresis, the raw data obtained could be analyzed using GeneMapper ID-X v1.6 software to obtain a graphical representation of the DNA profile called an electropherogram (EPG) (This same method was used in our previous study^18^).

#### Genetic profile analysis

On genetic profile, each detected allele is represented by a peak whose height is measured in rfu (relative fluorescence unit). According to an internal French Gendarmerie validation process (using a DNA source of known quantity and known STR profile, amplifications at 28 cycles were carried out by decreasing the initial DNA quantity until a genetic profile with a true allele without artifacts was obtained), the detection threshold (to avoid background noise) was set at 50 rfu.

A reference genetic profile was established prior to experiments. Each genetic profile of the samples used was compared with the reference genetic profile. This preliminary study made it possible to characterize a homozygous and heterozygous marker. If an allele is detected with a fluorescence intensity above 400 rfu, then the marker is considered homozygous. A marker is said to be heterozygous if the fluorescence intensity is greater than 150 rfu. The different genetic profiles were evaluated according to several criteria. A genetic profile was declared usable if the number of amplified STRs was greater than 5 (according to the recommendations of the French database FNAEG: Fichier National Automatisé des Empreintes Génétiques). The presence of both the Q and S quality sensors showed the good efficiency of the PCR.

### Statistical analysis

The various results obtained are presented as the mean ± standard error of several independent experiments. Several types of statistical analysis were used:

- To check whether the distribution was normal, a Shapiro–Wilk normality test was performed for all experiments. Subsequently, a one-way analysis of variance (ANOVA) was used to assess differences, using GraphPad Prism® software (Dotmatics, USA).
- Principal Component Analysis (PCA) was used to study multidimensional data sets with quantitative variables. PCA yielded a set of principal components (PCs) that explained the greatest variability in these data sets. This analysis was thus able to highlight characteristics and their correlation with the physicochemical properties of the sample. This analysis was carried out using XLSTATS software (Lumivero, USA).

### Supplementary Information

Supplementary Information.

## Data Availability

The various STR markers analyzed in this study are available in supplementary data (Table S1) and in the GenBank® database on the website [https://www.ncbi.nlm.nih.gov/genbank ]. For the raw data (in accordance with the ethical approval of the PJGN (Pôle Judiciaire de la Gendarmerie Nationale, recommendation n°10 of 12/03/2021) some restrictions apply to the availability of these data, which are not accessible to the public, because they involve sensitive data (genetic identification). They are stored in a secure database owned to the French Gendarmerie Nationale. They are available from the corresponding authors on reasonable request and with the authorization of the PJGN ethics committee.
