# Supplementary material for: Detection of invisible biological traces in relation to the physicochemical properties of substrates surfaces in forensic casework
Source: Sci Rep. 2024 Jun 10;14:13271. doi: 10.1038/s41598-024-63911-1 (PMC11164948; doi:10.1038/s41598-024-63911-1)
Supplement: Supplementary file 1 — Supplementary Information. [file 41598_2024_63911_MOESM1_ESM.docx]

**Supplementary Data: Detection of invisible biological traces in relation to the physicochemical properties of substrates surfaces in forensic casework.**


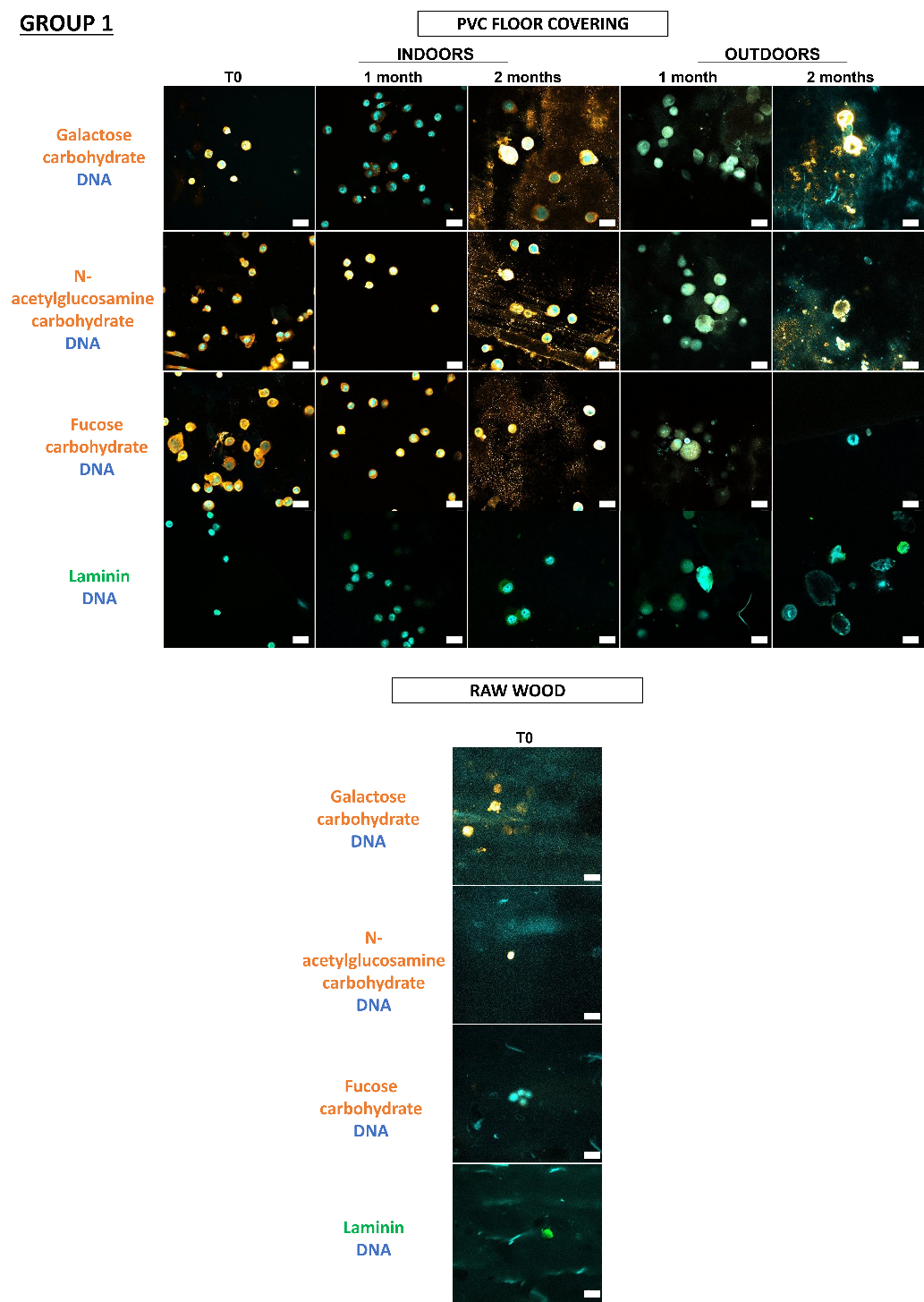


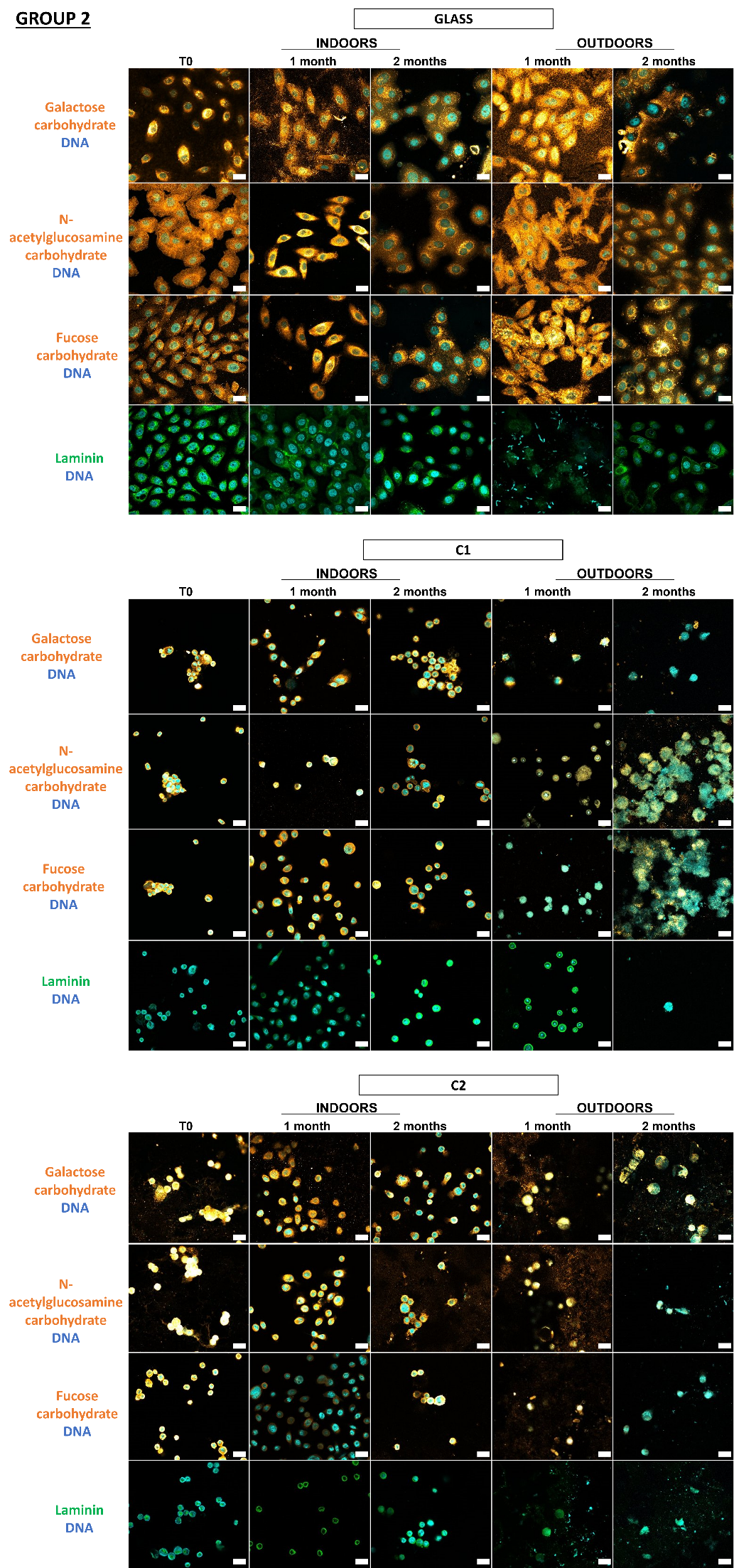


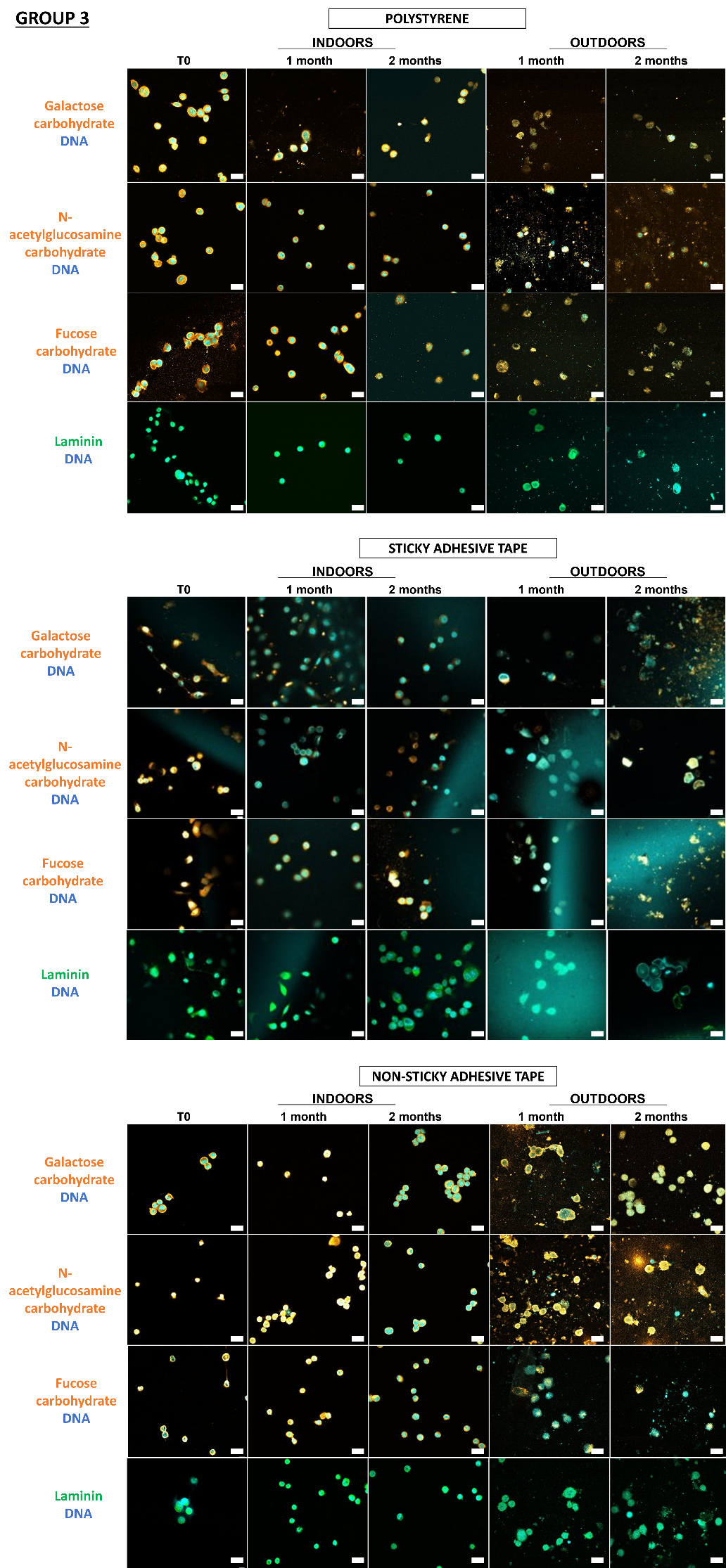


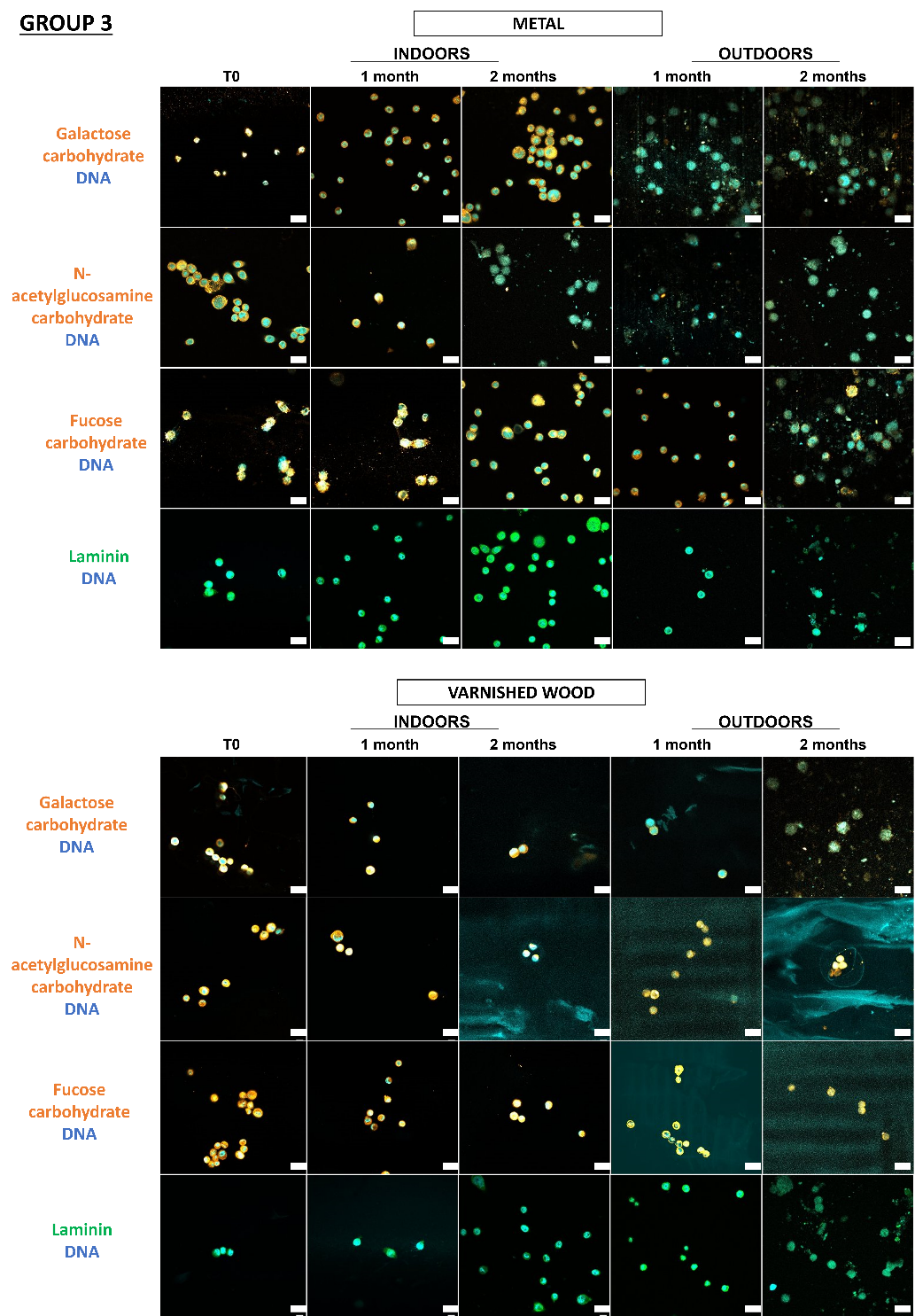


**Figure S1: Detection and persistence of lectin, antibody, and DNA targets on keratinocyte cells on the panel of substrates.**

5000 keratinocyte cells were seeded on 10 substrates. These 10 substrates were divided into 3 groups according to the principal component analysis of their physicochemical characteristics: group 1 (PVC flooring, raw wood), group 2 (glass, C1, C2), group 3 (polystyrene, sticky adhesive tape, non-sticky adhesive tape, metal, varnished wood). Substrates were placed indoors or outdoors for 2 months. Images showed keratinocyte cells visualized by confocal microscopy. For raw wood, after 1 month the cells could not be visualized due to the fibrous nature of the substrate. Cells were incubated in the presence of PNA, SNA and UEA lectins (orange), which recognize galactose, N-acetylglucosamine and fucose carbohydrates respectively, or labeled with an antibody specifically directed against laminin (green)DNA from nuclei was stained with Hoechst 33342 (Blue). Images are representative data from three independent experiments. Scale bar: 10 µm.


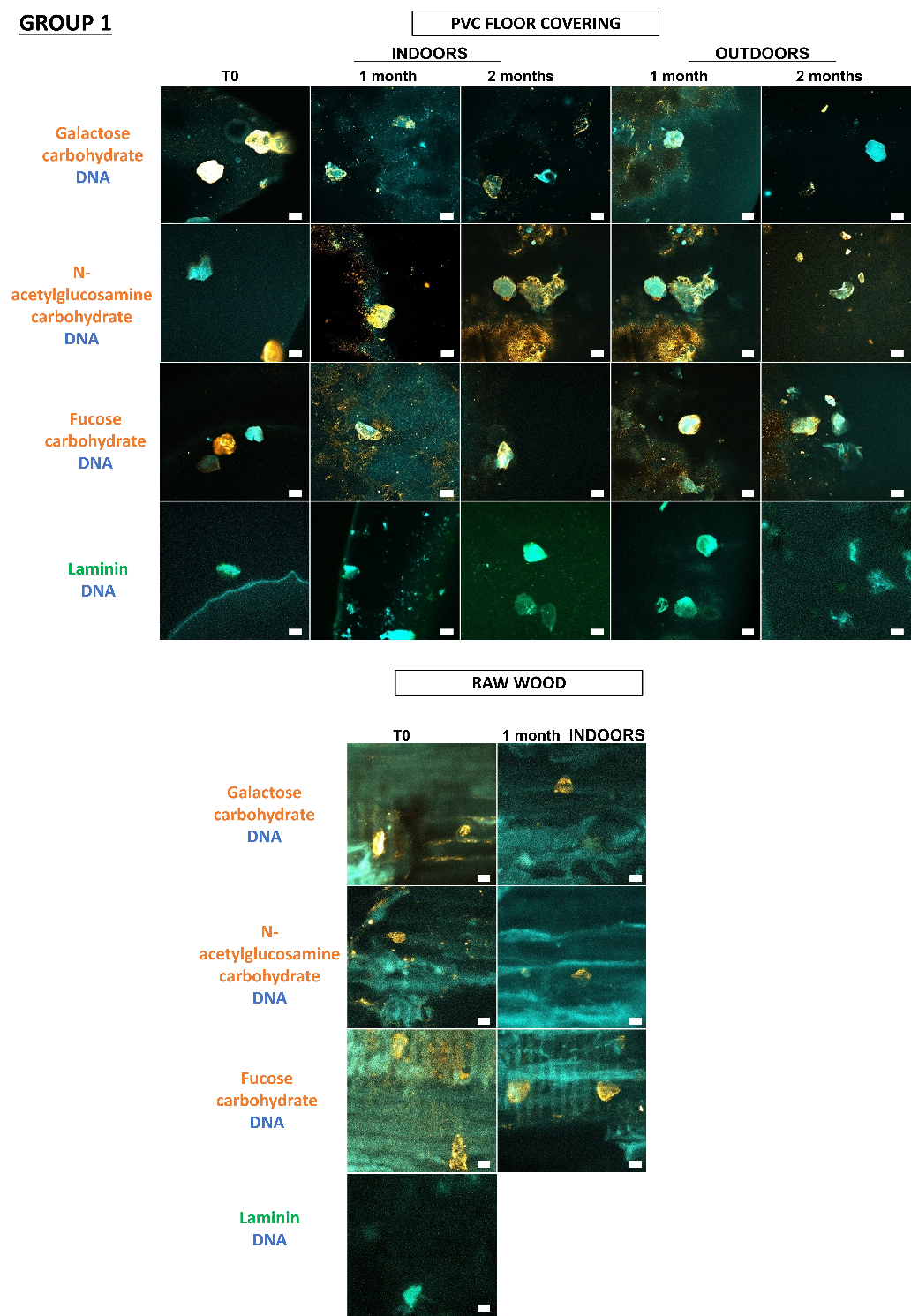


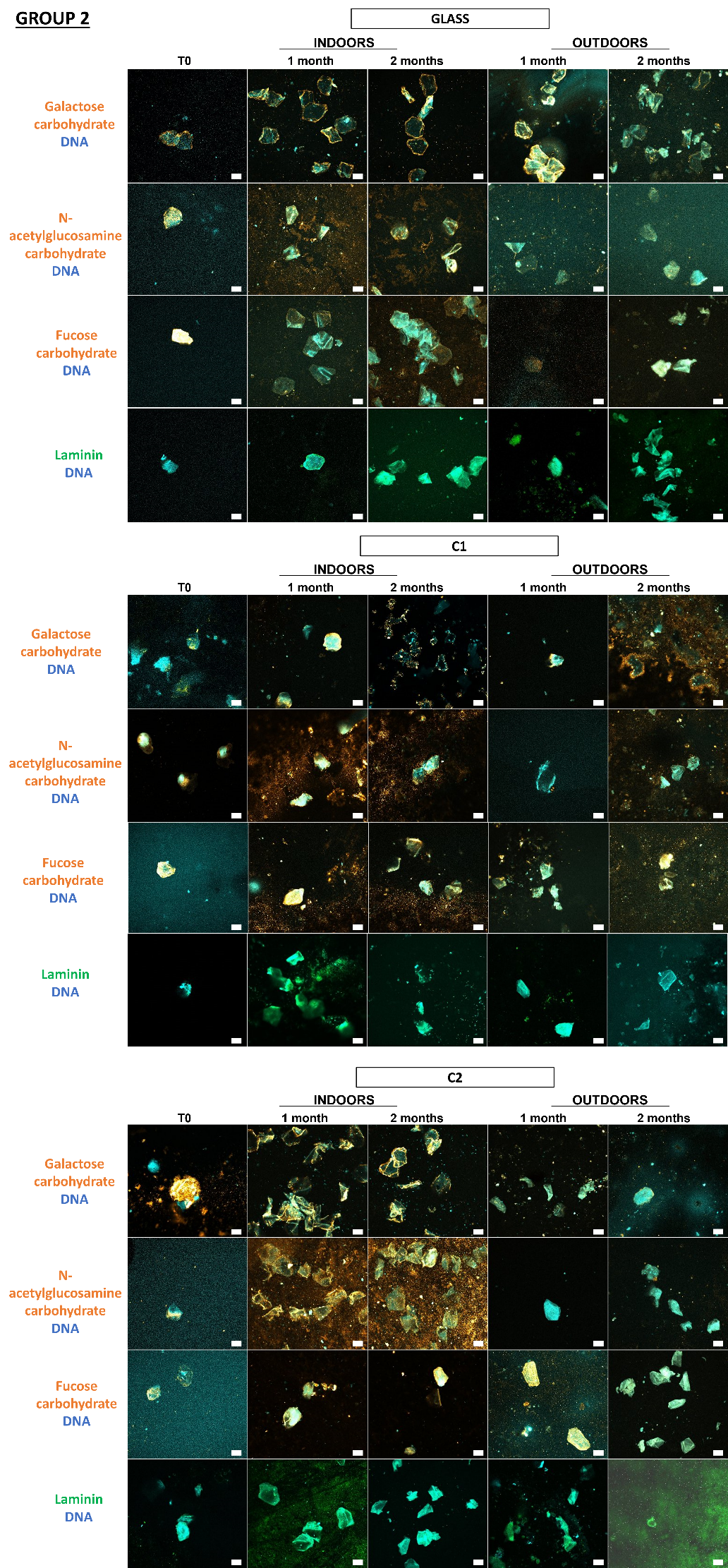


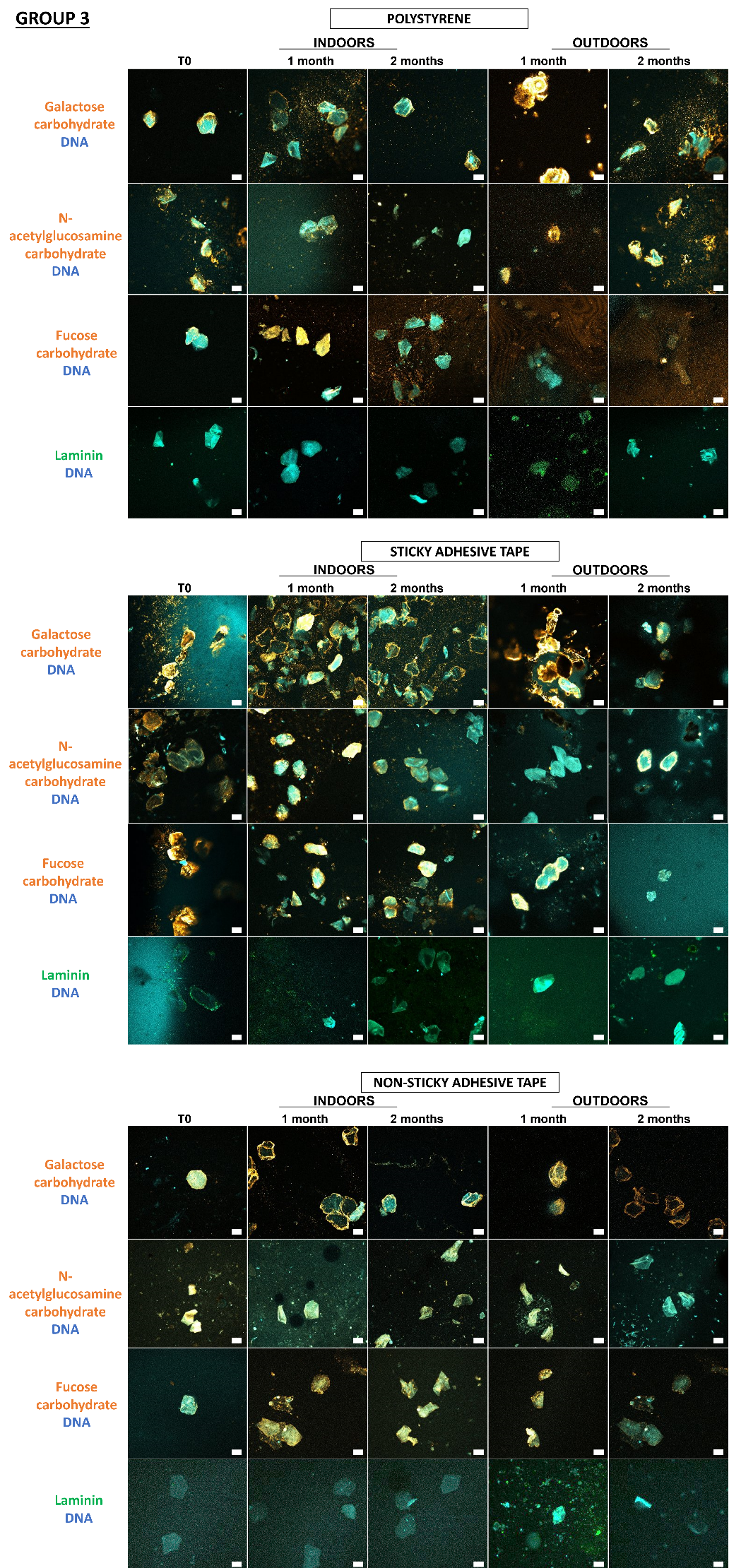


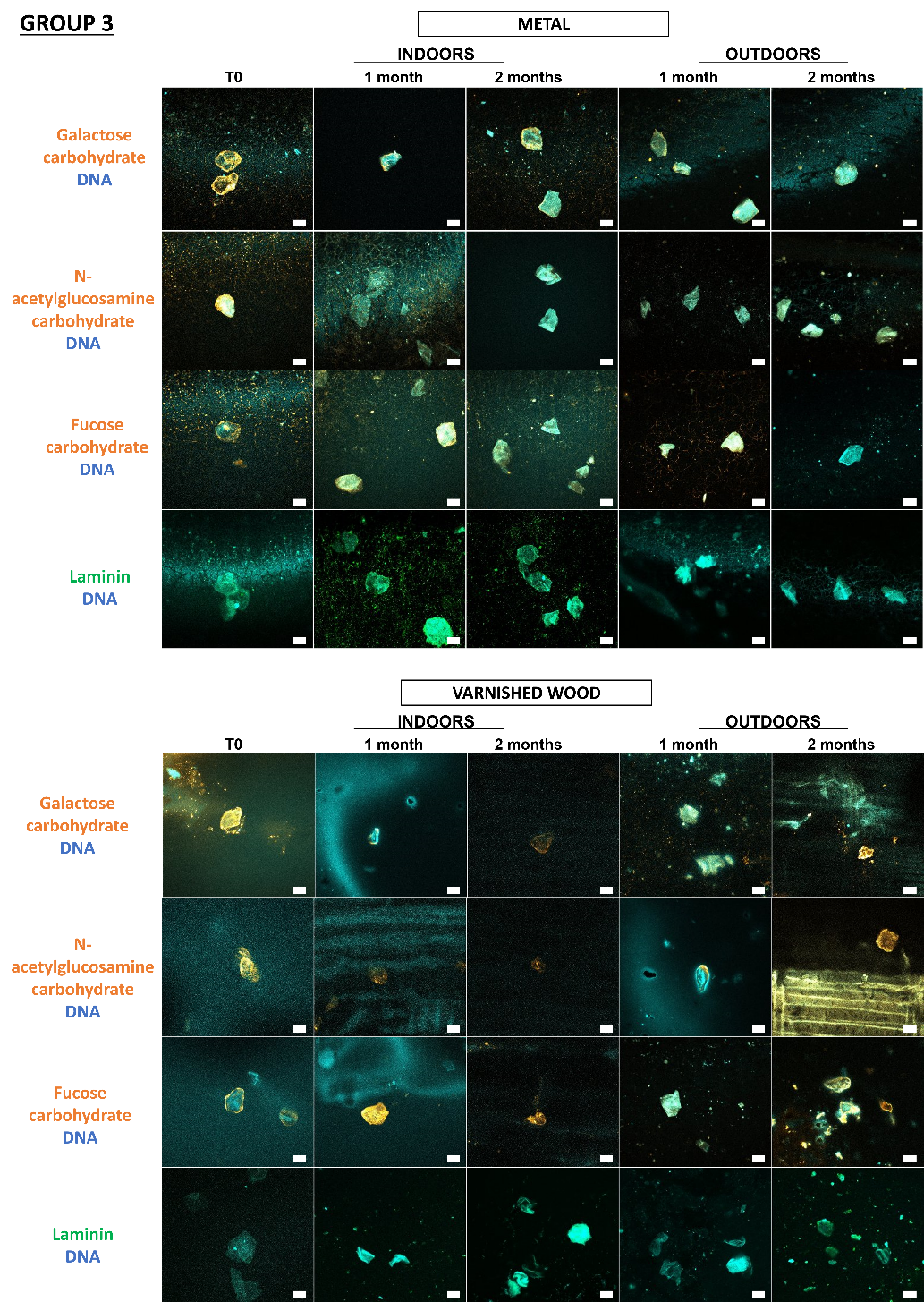


**Figure S2: Detection and persistence of lectin, antibody, and DNA targets on fingermarks on the panel of substrates.**

Fingermarks were deposited on 10 substrates for 10 seconds. These 10 substrates were divided into 3 groups according to the principal component analysis of their physicochemical characteristics: group 1 (PVC flooring, raw wood), group 2 (glass, C1, C2), group 3 (polystyrene, sticky adhesive tape, non-sticky adhesive tape, metal, varnished wood). Substrates were placed indoors or outdoors for 2 months. Images showed keratinocyte cells visualized by confocal microscopy. For raw wood, after 1 month indoors, cells could not be visualized due to the fibrous nature of the substrate. Cells were incubated in the presence of PNA, SNA and UEA lectins (orange), which recognize galactose, N-acetylglucosamine and fucose carbohydrates respectively, or labeled with an antibody specifically directed against laminin (green)DNA from nuclei was stained with Hoechst 33342 (Blue). Images are representative data from three independent experiments. Scale bar: 10 µm.

| Locus | Registration number GenBank® | Reference allele repeat motif |
| --- | --- | --- |
| Amelogenin X | M55418 | - |
| Amelogenin Y | M55419 | - |
| DYS391 | AC011302 | [TCTA]11 |
| D1S1656 | NC_000001.9 | [TAGA]16 [TGA][TAGA][TAGG]1[TG]5 |
| D2S441 | AL079112 | [TCTA]12 |
| D2S1338 | G08202 | [TGCC]6[TTCC]11 |
| D3S1358 | 11449919 | TCTA [TCTG]2 [TCTA]15 |
| D5S818 | G08446 | [AGAT]11 |
| D7S820 | G08616 | [GATA]12 |
| D8S1179 | G08710 | [TCTA]12 |
| D10S1248 | AL391869 | [GGAA]13 |
| D12S391 | G08921 | [AGAT]5 GAT [AGAT]7 [AGAC]6 AGAT |
| D13S317 | G09017 | [TATC]13 |
| D16S539 | G07925 | [GATA]11 |
| D18S51 | L18333 | [AGAA]13 |
| D19S433 | G08036 | AAGG [AAAG] AAGG TAGG [AAGG]11 |
| D21S11 | AP000433 | [TCTA]4 [TCTG]6 [TCTA]3 TA [TCTA]3 TCA [TCTA]2 TCCATA [TCTA]11 |
| D22S1045 | AL022314 | ATT]14 ACT [ATT]2 |
| CSF1PO | X14720 | [AGAT]12 |
| FGA (FIBRA) | M64982 | [TTTC]3 TTTTTTCT [CTTT]13 CTCC [TTCC]2 |
| SE33 (ACTBP2) | NG000840 | [AAAG]9 AA [AAAG]16 |
| TH01 (TC11) | D00269 | [TCAT]9 |
| TPOX | M68651 | [AATG]11 |
| vWA | M25858 | TCTA [TCTG]4 [TCTA]13 |

**Table S1: Locus-specific information for Investigator 24plex kits.**


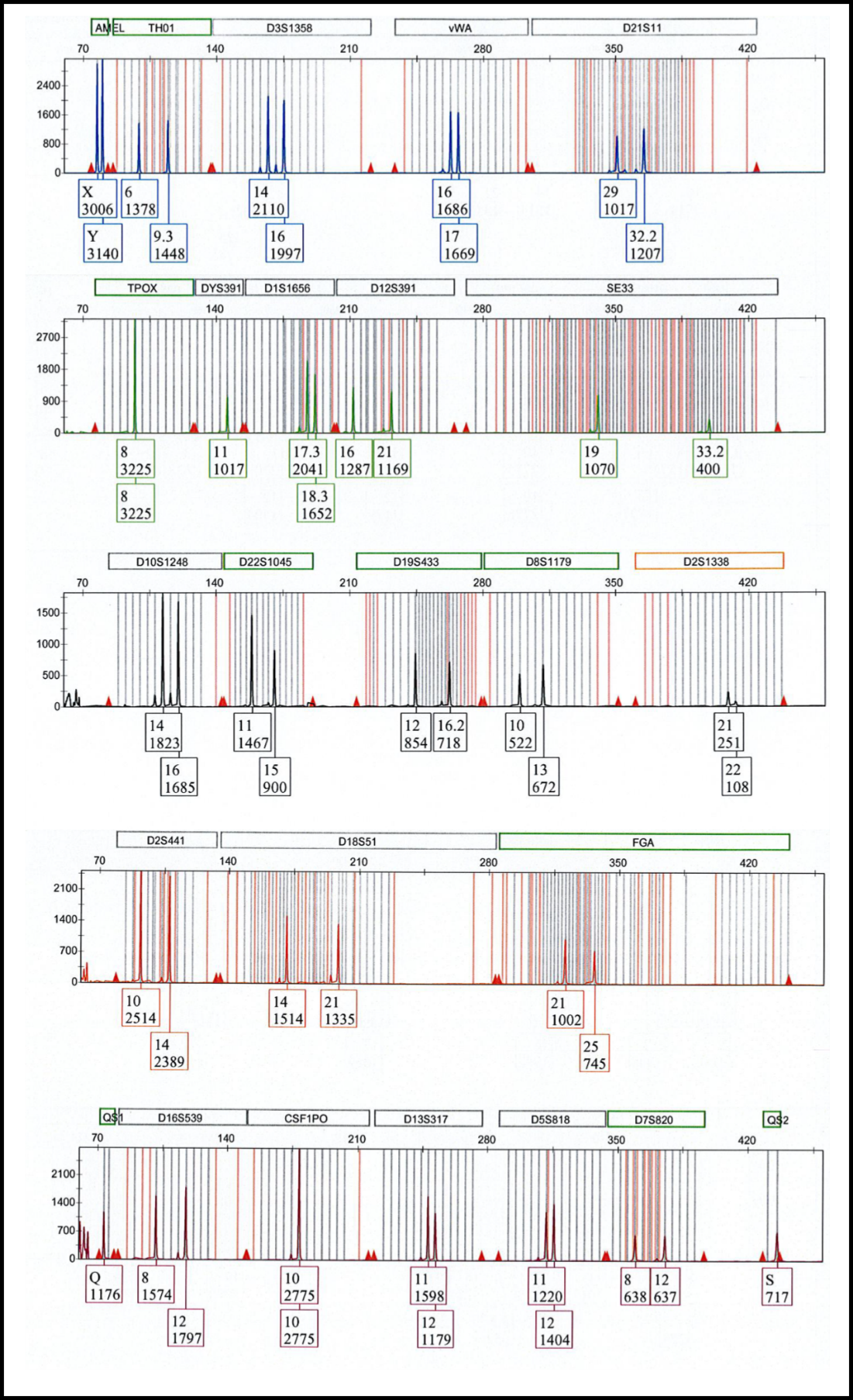


**Figure S3: Example of a genetic profile of keratinocytes on sticky adhesive tape.**

75 keratinocytes were deposited on the substrate and collected using a microFLOQ®. Amplification was performed using Investigator 24plex QS. Human gender determination was performed by analyzing the amelogenin gene which is common to both X and Y chromosomes. If the two quality sensors (Q and S) were visible on the electrophoregram, it meant that the PCR was successful and there was no inhibition. Results are representative of at least two independent experiments.
